# Toward Standardized Microscale Tensile Testing for Two‐Photon Polymerization‐Fabricated Materials in Liquid

**DOI:** 10.1002/smsc.202500228

**Published:** 2025-07-22

**Authors:** Grayson Minnick, Timothy Goldsmith, Bahareh Tajvidi Safa, Amir Ostadi Moghaddam, Jordan Rosenbohm, Nickolay V. Lavrik, Wei Gao, Ruiguo Yang

**Affiliations:** ^1^ Department of Mechanical and Materials Engineering University of Nebraska‐Lincoln Lincoln NE 68588 USA; ^2^ Department of Biomedical Engineering Michigan State University East Lansing MI 48824 USA; ^3^ Center for Nanophase Materials Sciences Oak Ridge National Laboratory Oak Ridge TN 37831‐6054 USA; ^4^ J. Mike Walker ’66 Department of Mechanical Engineering Texas A&M University College Station TX 77843 USA; ^5^ Department of Materials Science and Engineering Texas A&M University College Station TX 77843 USA; ^6^ Institute for Quantitative Health Science and Engineering Michigan State University East Lansing MI 48824 USA

**Keywords:** mechanical characterization, tensile testing, two‐photon polymerization

## Abstract

Two‐photon polymerization (TPP) enables the fabrication of intricate 3D microstructures with submicron precision, offering significant potential in biomedical applications like tissue engineering. In such applications, to print materials and structures with defined mechanics, it is crucial to understand how TPP printing parameters impact the material properties in a physiologically relevant liquid environment. Herein, an experimental approach utilizing microscale tensile testing (μTT) for the systematic measurement of TPP‐fabricated microfibers submerged in liquid as a function of printing parameters is introduced. Using a diurethane dimethacrylate‐based resin, the influence of printing parameters on microfiber geometry is first explored, demonstrating cross‐sectional areas ranging from 1 to 36 μm^2^. Tensile testing reveals Young's moduli between 0.5 and 1.5 GPa and yield strengths from 10 to 60 MPa. The experimental data show an excellent fit with the Ogden hyperelastic polymer model, which enables a detailed analysis of how variations in writing speed, laser power, and printing path influence the mechanical properties of TPP microfibers. The μTT method is also showcased for evaluating multiple commercial resins and for performing cyclic loading experiments. Collectively, this study builds a foundation toward a standardized microscale tensile testing framework to characterize the mechanical properties of TPP printed structures.

## Graphical abstract

A high‐throughput microscale tensile testing (μTT) device is reported to perform mechanical characterization of TPP‐printed microfibers in liquid environments. The device consists of arrays of μTT structures printed from biocompatible photoresins and examines the effect of varied printing parameters on microfibers’ mechanical properties. The methodology can be adopted as a standard technique for mechanical testing of TPP printed structures.

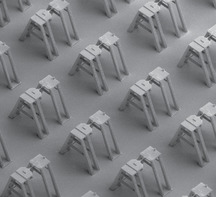

## 1 Introduction

Since its inception,^[^ ^1^ ^]^ two‐photon polymerization (TPP), or multi‐photon polymerization, has steadily advanced as a transformative technology in 3D fabrication.^[^ ^2^, ^3^, ^4^, ^5^, ^6^ ^]^ TPP has demonstrated applications across diverse fields, such as optics,^[^ ^7^, ^8^, ^9^, ^10^, ^11^ ^]^ electronics,^[^ ^12^, ^13^, ^14^ ^]^ microfluidics,^[^ ^15^, ^16^ ^]^ mechanobiology,^[^ ^7^, ^17^, ^18^, ^19^ ^]^ and material sciences.^[^ ^4^, ^20^, ^21^, ^22^, ^23^ ^]^ For instance, in micro‐optics, TPP facilitates the creation of intricate, multilens systems,^[^ ^24^ ^]^ complex lens geometries,^[^ ^25^ ^]^ and microlenses tailored for biological applications.^[^ ^26^ ^]^ In microelectronics, TPP supports the fabrication of conductive materials,^[^ ^12^, ^13^, ^14^ ^]^ facilitating the development of complex microdevices with applications in sensor technologies.^[^ ^27^ ^]^ In mechanobiology, TPP has enabled the design of bio‐scaffolds^[^ ^28^ ^]^ for the study of cellular behaviors such as contraction^[^ ^29^ ^]^ and adhesion.^[^ ^30^, ^31^ ^]^ More recently, 4D microprinting has emerged,^[^ ^6^, ^32^, ^33^, ^34^ ^]^ allowing for structures to dynamically respond to environmental stimuli. TPP's utility also extends to the design of micro‐metamaterials,^[^ ^35^, ^36^ ^]^ microrobotics,^[^ ^37^, ^38^ ^]^ and flexible surface structuring.^[^ ^39^ ^]^ As these utilities evolve, ongoing work aims to improve both printing resolution^[^ ^40^, ^41^ ^]^ and scalability.^[^ ^42^, ^43^ ^]^ Innovations in multifocus printing^[^ ^44^, ^45^, ^46^ ^]^ and processing optimization^[^ ^42^ ^]^ are refining TPP's precision to allow for the production of macroscale, microstructured objects with reduced processing time. Specifically, grayscale TPP, or grayscale lithography, utilizes dynamically modulated laser power to provide higher resolutions, especially for curved or lens geometries.^[^ ^47^ ^]^ These recent advancements are paving the way for the widespread adoption of TPP in both research and industrial applications, where scale, speed, and material integrity are crucial.

TPP's potential in advanced material fabrication presents a critical need for reliable mechanical testing to both assess material properties and establish standardized benchmarks. Early approaches to TPP mechanical testing employed a range of methods, including indirect force measurement,^[^ ^48^ ^]^ microdeformation analysis of cantilevers^[^ ^49^, ^50^, ^51^, ^52^ ^]^ and microbeams,^[^ ^29^ ^]^ and indentation.^[^ ^53^, ^54^, ^55^, ^56^, ^57^, ^58^, ^59^ ^]^ While these methods offer valuable insights, they are constrained by limitations in measurement techniques and their reliance on nonbiological testing environments. More recently, these approaches have expanded to encompass Raman spectroscopy,^[^ ^42^, ^54^, ^55^, ^58^ ^]^ allowing for more correlative atomic bonding information, such as the degree of conversion, to better understand material behavior. Recent studies in microscale evaluation, such as tensile and compression testing in SEM environments,^[^ ^42^, ^56^, ^57^ ^]^ have highlighted the importance of a direct and comprehensive mechanical characterization.

Within this framework, tensile testing has emerged as a key method for directly measuring the mechanical response of TPP‐fabricated materials.^[^ ^42^, ^56^, ^57^, ^60^ ^]^ Historical developments in tensile testing, from electrospun nanofibers^[^ ^61^ ^]^ to MEMS‐based devices,^[^ ^62^, ^63^ ^]^ have laid the groundwork for precise mechanical assessments; however, environmental and throughput limitations persist. These methods fall short of testing within a biologically relevant environment which is essential for TPP applications, as the organic materials produced can be highly susceptible to liquid absorption or penetration. To address these limitations, we recently introduced a novel TPP microtensile tester (μTT).^[^ ^64^ ^]^ Using the TPP μTT, we identified some interesting material behaviors, including shape recovery after large‐strain deformation. We also observed hydration‐dependent mechanical properties, with microfibers showing greater strength when dry and increased compliance when hydrated. These observations underscore the importance of testing TPP materials designed for tissue engineering under biologically relevant conditions. Additionally, in contrast to region‐specific testing methods, such as nanoindentation, microtensile testing provides a direct measurement of classical mechanical properties to achieve a complete mechanical characterization profile. Recent studies have demonstrated that TPP‐fabricated structures can support cellular invasion and proliferation.^[^ ^43^, ^65^, ^66^, ^67^ ^]^ To better design materials that replicate native tissue properties, there is a clear need to test TPP‐fabricated materials in environments that closely mimic in vivo conditions, where material properties and geometry directly influence cellular response and tissue maturation.^[^ ^19^, ^68^ ^]^ In light of the push toward standardized testing protocols^[^ ^42^ ^]^ and the need for biologically relevan*t* testing conditions, our approach aims to provide a standardized microscale tensile testing method for TPP fabricated materials.

In this article, we present an enhanced version of the μTT aimed toward a standardized tensile testing methodology for TPP printed microstructures. This enhanced approach integrates comprehensive testing protocols and advanced analysis techniques to provide a deeper understanding of the relationship between processing parameters and the fiber's mechanical properties. Key parameters, including writing power, writing speed, and hatching patterns, were systematically studied to understand their impact on fiber structure and performance. These experiments were conducted under physiologically relevant liquid conditions, where the polymers’ mechanical behavior is strongly influenced by factors such as hydration and liquid absorption. Our mechanical testing data can be accurately described by an Ogden hyperelastic polymer model which captures the large‐strain nonlinear behavior of the materials tested. Using the model to evaluate material properties, we examine the influence of strain rate, writing power, writing speed, and hatching style. Furthermore, we demonstrate the device's multimaterial testing capabilities and cyclic testing functionality, highlighting its versatility for future applications.

## 2 Results and Discussion

### 2.1 Two‐Photon Polymerization Process Considerations

**Figure 1:**
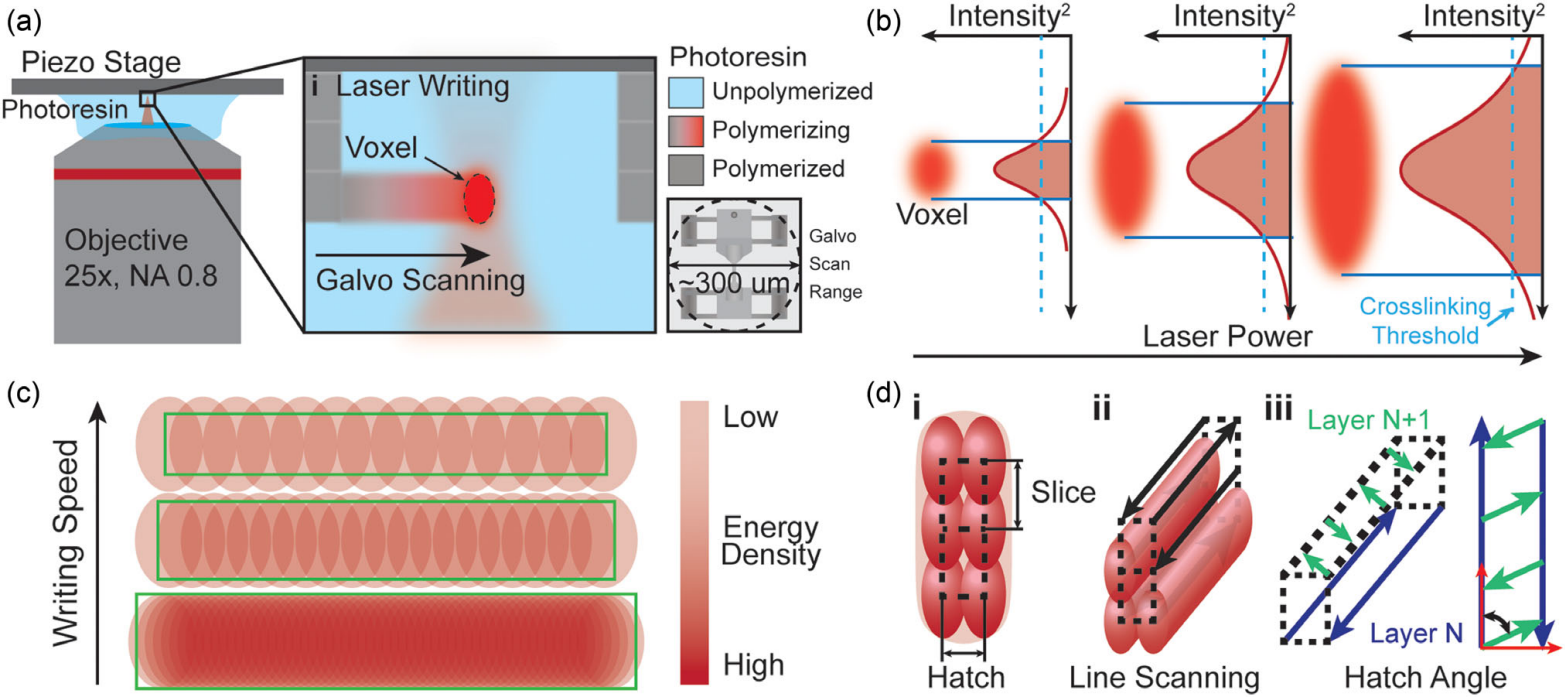
Two‐photon polymerization processes and parameters description. a) Dip‐in Laser (DiL) two‐photon polymerization (TPP). i) The focused femtosecond pulse generates a voxel (volumetric pixel). The XY laser scanning is controlled through galvo mirrors with a range of ≈300 μm. b) Geometric voxel power scaling. Visual description of the increase in voxel size with increasing laser power. c) Writing speed and volumetric energy delivery. Visual description of the increase in energy delivered to volumes at lower writing speed. d) Writing parameter definitions. i) Slice and hatch distance description. Slice describes the distance between consecutive layers. Hatch describes the distance between consecutive scans of one layer. ii,iii) Line scanning and hatch angle describe the writing path and angle between consecutive layers, respectively.

The TPP process is highly dynamic and requires precise control over technique and parameters. The main input parameters include writing power, writing (or scan) speed, slicing and hatching distance, and writing path. These parameters contribute to the degree of conversion (DC) and crosslinked network of polymer materials. **Figure** **1** provides an overview of these writing processes and their effects on voxel geometry and observed material outcomes. A TPP dip‐in laser configuration (Figure 1a) was used in this study. Briefly, the TPP setup employs an acousto‐optic modulator to control beam power and galvo scanning mirrors to adjust the XY voxel position and scan speed through an inverted objective “dipped” into the photoresin. The TPP μTT structures in this study were produced within a single galvo‐scanning region (Figure 1a(i)), allowing for continuous layer‐by‐layer material fabrication.

The femtosecond laser pulse responsible for TPP represents a geometric instance of energy with a Gaussian distribution. Here, the energy density (or volumetric intensity) drives molecule absorption and initiates radical generation within the photoresin. This radical generation triggers covalent bonding, forming the material structure. The degree of crosslinking (or DC), a critical factor in material formation, is directly linked to the energy absorbed in the focused voxel region. When excess energy is absorbed, it initiates radical diffusion, where radical propagation expands from the voxel region, resulting in larger material geometry. Overexposure and the resulting geometric expansion signify a high covalent bond density within the nuclear region, quantified as the DC. To envision a model of the nanostructure, consider a randomly oriented, energy‐minimized molecular simulation of the molecules which form the resin; here, bonding potentials are identified. The energy delivered to a specific volume is absorbed at less‐than‐ideal efficiency by the photoinitiator. This determines the percentage of these bonding potentials that will be realized in bonds, resulting in a material with varying degrees of crosslinking and, consequently, stiffness. Therefore, by precisely controlling material crosslinking, a material's mechanical behavior can be finely tuned. This work presents a method for evaluating the geometric and mechanical effects of TPP parameter selection, enabling precise tuning of material specifications.

As visualized in Figure 1b, voxel geometry increases with writing power. Higher power raises the peak intensity, broadening the region exceeding the polymerization threshold and resulting in a larger and more crosslinked voxel.^[^ ^3^, ^54^, ^69^, ^70^ ^]^ Additionally, the increased energy density can induce radical diffusion from the voxel's focus, further contributing to the expansion of size and DC. Writing speed, in contrast, does not alter voxel geometry but impacts target geometry by increasing volumetric energy delivery through voxel overlap, as illustrated in Figure 1c. Slower writing speeds result in greater volumetric energy delivery, leading to higher DC. With higher DC, excess energy promotes radical diffusion, further contributing to larger geometries.^[^ ^3^ ^]^ This interplay between volumetric energy delivery and crosslinking density extends to line‐scan parameters and patterns, as shown in Figure 1d. Line‐scan parameters, such as slicing distance, hatching distance, and hatching angle, control energy delivery without altering individual voxel geometry. Their role in distributing energy across the material introduces a critical factor in structuring crosslinking patterns. By influencing the spatial arrangement and overlap of crosslinked regions, these parameters contribute to material properties in a unique and controlled manner.

In our fabrication process, the TPP μTT structure was printed first, with consistent processing parameters and material (IP‐S) to ensure uniform mechanical properties across batches. Subsequently, the barbell testing structure was crosslinked onto the μTT structure, connecting the force and sensing components to enable actuation, transmission, and force sensing. This two‐step printing method enables testing of unique material properties without altering the sensing structure properties. Print anomalies were uncommon and removed if visually discernable. This approach is demonstrated using multiple crosslinking materials, including IP‐S, IP‐Dip, IP‐Visio, and IP‐PDMS.

### 2.2 Influence of Writing Parameters on Fiber Dimensions

**Figure 2:**
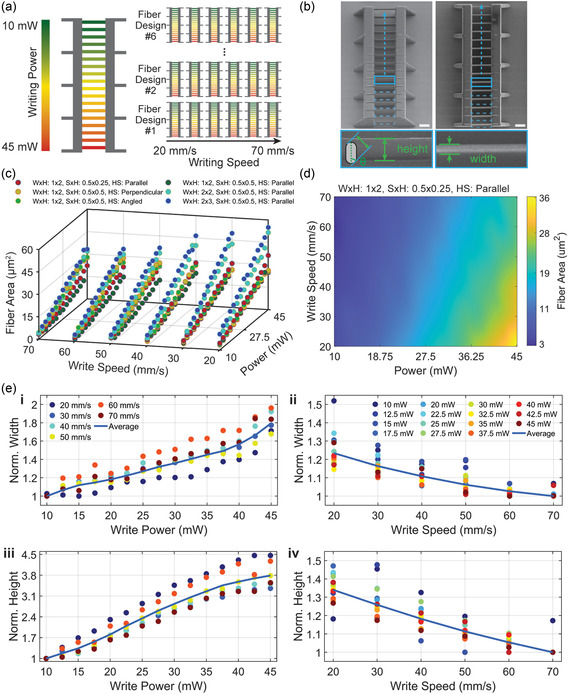
Evaluation of fiber geometry with varying process parameters. a) Overview of the fiber bridge study showing six different IP‐S fiber design variants, printed across a range of writing speeds (20–70 mm s^−1^) and powers (10–45 mW). Fiber bridges are arranged in arrays for systematic parameter evaluation. b) SEM images of the fiber cross‐sections used for area analysis. Measurements of width and height were taken from each cross‐section, with the area calculation scheme described previously.^[^ ^64^ ^]^ c) Fiber area results for each combination of writing power and speed across all fiber designs. Increased power and decreased speed generally resulted in larger fiber areas, with some variations observed based on fiber design. d) Heat map illustrating the relationship between fiber area, writing speed, and power for a parallel line‐scanned fiber design (W × H: 1 × 2, *S* × H: 0.5 × 0.25, HS: parallel). e) Normalized fiber i,ii) width and iii,iv) height evaluations as a function of i,iii) write power and ii,iv) write speed for a parallel line‐scanned fiber design (W × H: 1 × 2, *S* × H: 0.5 × 0.25, HS: parallel). Normalization highlights the relative impact of each parameter, with power showing a greater influence on geometry than speed.

We first evaluated the influence of power, speed, slice, hatch, and design parameters on fiber geometry. Parameter‐varied bridge fiber arrays, as shown in **Figure** **2**a, were printed and analyzed using SEM to determine cross‐sectional areas (Figure 2b). Six different fiber design variants were tested across 6 writing speeds with 15 power variations each, resulting in 90 measurements per fiber design (slice × hatch, hatch style). These results provide insight into the relationship between processing parameters and physical fiber geometry. The material used in the presented study was IP‐S, a reliable photoresist supplied by Nanoscribe.

As expected, increased power and decreased writing speed generally resulted in larger fiber geometries (Figure 2c,d).^[^ ^56^, ^71^ ^]^ However, an exception was observed for perpendicular and angular hatched fibers, where higher writing speeds produced wider fibers (Figure S7 and S8, Supporting Information). This anomaly is likely due to overshoot effects caused by the rapid back‐and‐forth line scanning of short perpendicular patterns. In these cases, the perpendicular line scan components of the fibers are particularly short, and the fast scan speed dynamics result in geometries extending slightly beyond their intended target, effectively enlarging the fiber width. This unintended overshoot highlights the influence of scan dynamics on pattern precision and material geometry. It should be noted that the overshoot observed in this study may be system specific and may vary in other TPP printing systems. Newer systems with adaptive scan modes are better equipped to mitigate these effects.

Focusing on the parallel scanned fibers, we further examined the width and height measurements. Figure 2e displays the normalized fiber width (i and ii) and height (iii and iv), each relative to the minimum value. The data reveal that writing power (10–45 mW average, scaled by the Nanoscribe Photonic Professional GT reference power of 50 mW mean output) had a more substantial impact over the processing range on both width and height, with a maximum growth of 1.8× in width and 3.8× in height. In contrast, variations in writing speed (20–70 mm s^−1^) led to only a 1.25× increase in width and a 1.35× increase in height.

Collectively, the bridge testing method provided an efficient and high‐throughput approach to assess the effects of TPP processing parameters. This study demonstrates an adaptable and straightforward evaluation technique for various photocompatible materials. This approach also allowed hands‐on exploration of the fabrication and control limits of the fibers. Future studies could use enhanced parameter scans to achieve target fiber geometries or apply spectroscopic microanalysis methods to evaluate the DC in a high‐throughput manner.

### 2.3 Microscale Tensile Testing

#### 2.3.1 Microscale Tensile Tester (μTT)

**Figure 3:**
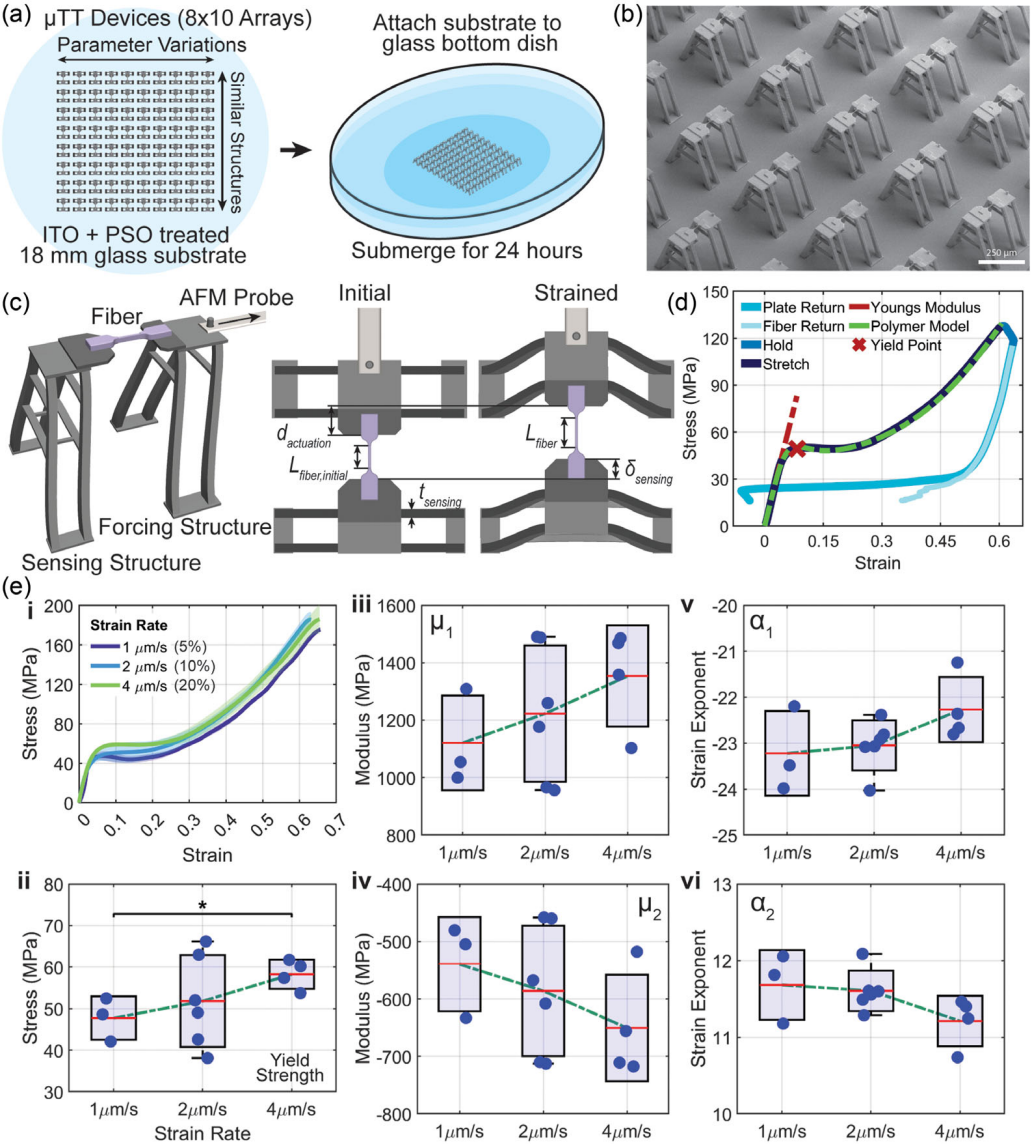
Microtensile testing (μTT) device setup and strain rate analysis. a) Substrate preparation for μTT devices from IP‐S, organized in an 8 × 10 array on a treated glass substrate. The substrate is attached to a glass‐bottom dish with transparent adhesive and submerged in water for 24 h to prepare for testing. b) SEM image of the μTT device array, showing the layout and structure of the testing devices. c) Diagram of the μTT device design, illustrating the forcing and sensing structures. A modified AFM probe actuates the structure and applies force to the fiber, creating a measurable strain. Initial and strained states depict the displacement parameters and fiber length changes used in the processing calculations. d) Representative stress–strain data from the μTT device showing the various stages of the testing process, including initial stretching, yield point, and hold. The stress–strain curve is fitted with an Ogden hyperelastic model. e) Comparison of stress–strain data and fit‐parameters at different strain rates of 1, 2, and 4 μm s^−1^ (*n* = 3, 6, and 4) corresponding to strain rates of 5%, 10%, and 20% (constant parameters; LP: 36 mW, W × H: 1 × 2 μm, S × H: 0.5 × 0.5 μm, HS: parallel). i) Representative engineering stress–strain curves; ii) yield stress $\sigma_{y}$; iii,iv) Ogden moduli, $\mu_{1}$and $\mu_{2}$; and v,vi) the Ogden strain exponents, $\alpha_{1}$and $\alpha_{2}$. In all panels, boxes represent mean ± standard deviation, whiskers indicate the minimum and maximum observed values, and dots are individual replicates.

The TPP μTT has been thoroughly detailed and documented previously.^[^ ^64^ ^]^ The device consists of two independent platforms: one for applying tensile strain (the forcing structure) and one for sensing force (the sensing structure) (**Figure** **3**). The sensing structures are calibrated for stiffness using cantilever deflection measured by AFM. This stiffness is then used to calculate the sensing force based on deflection ($F_{\text{sens}}=k_{\text{sens}}\cdot \delta_{\text{sensing}}$). These structures can be geometrically tuned by scaling the beam thickness ($k_{\text{sens}}\propto t_{\text{sensing}}^{3}$),^[^ ^64^ ^]^ allowing optimization for a wide range of microscale materials. To demonstrate this versatility, the structures can be fabricated with stiffness as low as 50 nN μm^−1^, suitable for sensing cellular junctions between single cell pairs.^[^ ^30^ ^]^ Further details can be found in Supplementary Section 1, Supporting Information. The arrays are printed structure by structure, with sizes up to 8 × 10, and parameter variations across the columns (Figure 3a). It is worth noting that compared with the previous generation of the μTT device and other studies using tensile testing,^[^ ^56^ ^]^ the current version allows for the systematic variation of printing parameters in these structure arrays, which significantly enhances the number of samples that can be tested in one run and paves the way for automatic testing and evaluation in a liquid environment.

All materials in this study were tested under submerged conditions by attaching the structures to a glass‐bottomed petri dish and submerging them in water (Figure 3a). Additionally, the structures have demonstrated stability and tensile testing potential in other media, such as PBS, cell media, and ethanol. Before testing, the structures and fibers were allowed to fully saturate in the liquid. We have previously shown that air versus water testing conditions have a significant impact on measured stiffness.^[^ ^64^ ^]^

#### 2.3.2 Polymer Model and Data Fitting

In the fitted studies presented, a single large deformation straining event was applied, but fiber fracture was not typically observed (Figure 3d). The typical material curve observed showed large‐strain hyperelastic polymer behavior (Figure 3d,e), with an initial elastic region, a yielding region marked by a dip in force, and a final energy‐intensive strain region. To capture the observed nonlinear behavior across a wide range of strain states, a two‐term Ogden hyperelastic model was employed to fit the experiments

(1) $$\sigma =\sum_{k=1}^{2}\frac{2\mu_{k}}{\alpha_{k}}\left[\lambda^{\alpha_{k}}-{\left(\frac{1}{\sqrt{\lambda }}\right)}^{\alpha_{k}}\right]$$

Here, *σ* is the Cauchy stress under uniaxial tension, and λ=1+ε is the stretch ratio (with *ε* being engineering strain). The model parameters $\mu_{k}$ and $\alpha_{k}$ govern the stiffness contribution of each term in the strain energy function and the associated strain‐dependent nonlinear response, respectively.

Using this two‐term approach, the Ogden model accurately reproduced the nonlinear response observed in 239 IP‐S experiments, with an average *R* ^2^ = 0.9943 + 0.0148. Considering the physical mechanism of microfiber deformation, the polymer transitions from an elastic state stabilized by hydrogen bonding to a regime in which these bonds become disrupted. Upon bond disruption, the polymer chains gain greater mobility, enabling long‐chain rearrangements as well as the evacuation of ambient liquid from interstitial regions.

Within the tested strain rates (5–20% s^−^^1^), the mechanical response shows insignificant variations (Figure 3e), indicating minimal strain rate effects in this strain range. Nevertheless, the stress–strain curves demonstrate a mild difference in stress evolution with strain rates (Figure 3e(i)). The fitted Ogden parameters appear to demonstrate a weak trend with increasing magnitudes for $\mu_{1}$, $\mu_{2}$ and the yield strength also follows this trend, consistent with a viscoelastic stiffening effect (Figure 3e(ii–iv)). Meanwhile, the exponents $\alpha_{1}$ and $\alpha_{2}$ show a weak decrease in magnitude (Figure 3e(v,vi)). Although the overlaps in the data indicate no statistically significant differences, the observed patterns broadly support the idea that the material exhibits mildly greater stiffness and strength under faster deformation.

### 2.4 μTT Experiments

#### 2.4.1 Writing Power

**Figure 4:**
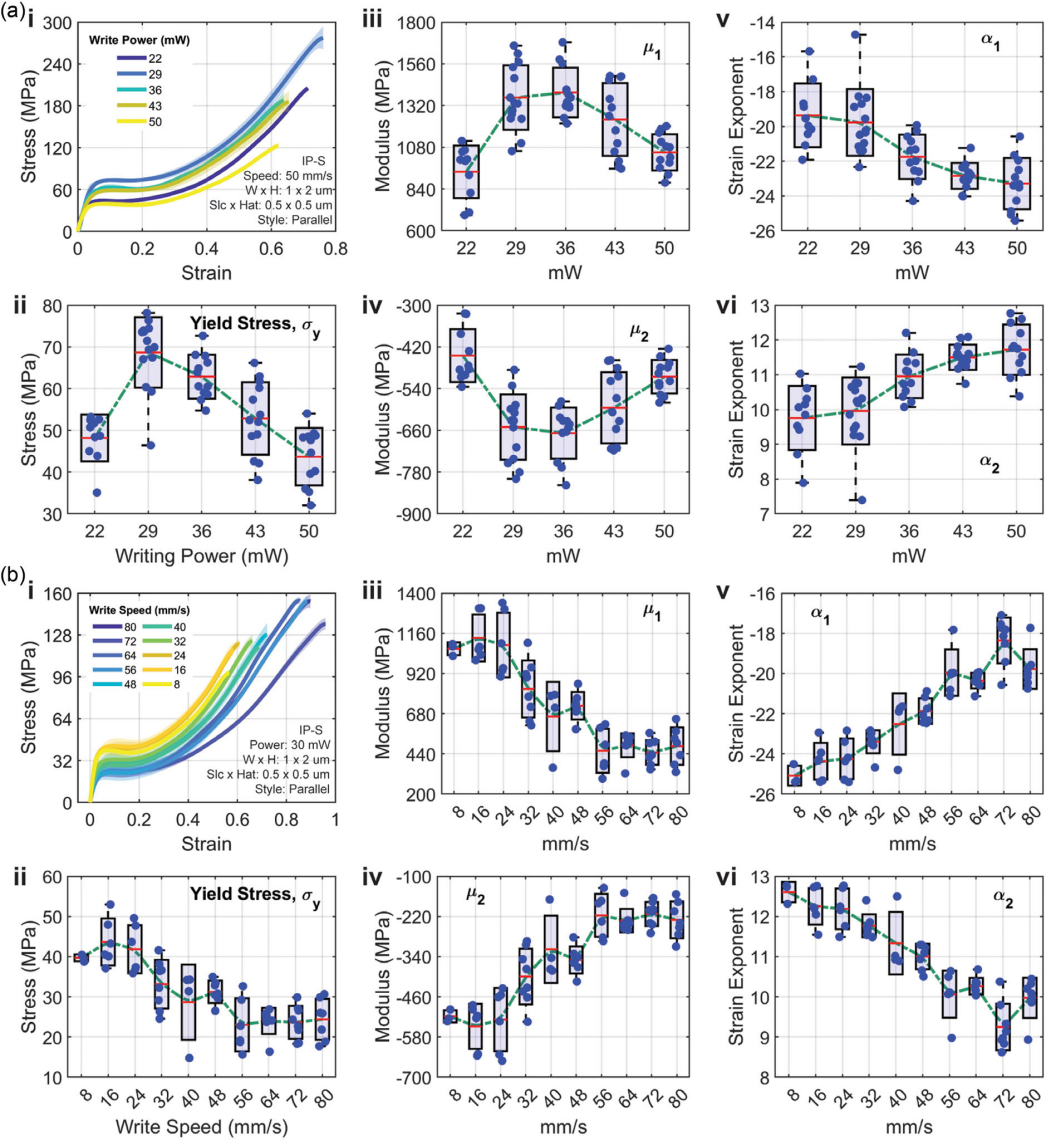
Mechanical properties and Ogden parameters of TPP‐printed IP‐S microfibers as a function of a) writing power and b) writing speed. (a) Mechanical properties of microfibers printed at writing powers of 22, 29, 36, 43, and 50 mW (*n* = 10, 14, 12, 13, 12), with constant parameters: WS = 50 mm s^−^^1^, W × H = 1 × 2 μm, S × H = 0.5 × 0.5 μm, style = parallel. i) Representative engineering stress–strain curves; ii) yield stress $\sigma_{y}$; iii,iv) Ogden moduli, $\mu_{1}$and $\mu_{2}$; and v,vi) Ogden strain exponents, $\alpha_{1}$and $\alpha_{2}$. (b) Mechanical properties of microfibers printed at writing speeds ranging from 8 to 80 mm s^−^^1^ (*n* = 3, 6, 6, 8, 4, 7, 6, 8, 8, 7), with constant parameters: power = 30 mW, W × H = 1 × 2 μm, S × H = 0.5 × 0.5 μm, style = parallel. i) Representative engineering stress–strain curves; ii) yield stress $\sigma_{y}$; iii,iv) Ogden moduli, $\mu_{1}$ and $\mu_{2}$; and v,vi) Ogden strain exponents, $\alpha_{1}$ and $\alpha_{2}$. In all panels, boxes represent mean ± standard deviation, whiskers indicate the minimum and maximum observed values, and dots are individual replicates.

In this study, the laser writing power varied between 44 and 100% with 5 power levels, corresponding to an average power range of 22–50 mW. Fibers were fabricated from IP‐S with a writing speed of 50 mm s^−1^, dimensions of 1 × 2 μm (W × H), slice and hatch distances of 0.5 × 0.5 μm (S × H), and a parallel hatching style. All fibers were printed on a single substrate, with two columns of fibers per power level. The results show a peak in fiber strength between 29 and 36 mW, as displayed in **Figure** **4**a. This trend continues for the magnitude of the modulus terms (Figure 4a(iii,iv)). Interestingly, at the highest power level (50 mW), the strength of the material is greatly reduced from the peak. This inverse relationship is attributed to the increase in fiber dimensions (Figure S3, Supporting Information), where the crosslink density is likely not uniform throughout the increased geometry, and this results in decreased strength measurements. The strain exponents show an increase in magnitude with increasing laser power (Figure 4a(v,vi)).

At higher power levels, the increased voxel size and energy density led to a significant enlargement of cross‐sectional geometry (Figure S3, Supporting Information). This enlargement reduced fiber strength by introducing nonuniform polymerization and structural inconsistencies, as excessive energy leads to uneven crosslinking density and radical diffusion. This examination highlights the influence of writing power on fiber mechanics, suggesting that an optimal power range exists where voxel size and energy density are balanced to maximize mechanical properties. These findings underscore the potential to fine‐tune writing power for fiber strength and geometry optimization.

#### 2.4.2 Writing Speed

Significant differences were observed across the range of writing speeds, underscoring the impact of energy exposure on the mechanical properties of the IP‐S fibers (Figure 4b). Fibers were fabricated at a power of 30 mW with dimensions of 1 × 2 μm (W × H), slice and hatch distances of 0.5 × 0.5 μm (S × H), and a parallel hatching style. Similar to the effects of varying writing power, an optimal balance between mechanical properties and geometry was identified, with peak performance occurring around 16 mm s^−1^ (Figure 4b(ii,iii)). Similar to power, SEM fiber area increases with decreased writing speed (Figure S4, Supporting Information), providing an explanation for the decrease in mechanical strength below 16 mm s^−1^. The first Ogden modulus exhibited substantial tunability, ranging from ≈440 to 1130 MPa, while yield strength varied from 23 to 44 MPa. These results demonstrate that the mechanical properties of the fibers can be finely controlled through optimization of the processing parameters. Writing speed does not affect the incident voxel geometry in comparison to writing power, highlighting its effectiveness as a tool for tunability. Adjusting writing speed is advantageous for achieving precise designs with specific DC and resulting mechanical properties. Furthermore, higher writing speeds correspond with more pronounced large‐strain deformation (Figure 4b(i)), a phenomenon well‐captured by the strain exponents, which decreased in magnitude with higher writing speeds (lower energy exposure), as shown in Figure 4b(v,vi).

Referring to the relationship between crosslinking and material behavior, this provides a clear example of how lower crosslinking density can lead to increased stretchability. This behavior can be attributed to the polymer's enhanced ability to reorient its constituent chains when not fully covalently crosslinked. This observation also highlights the potential for molecular‐scale modeling to enable a deeper multiscale understanding of polymer materials. Previous studies have shown correlation between writing parameters (laser power, writing speed, and slicing/hatching distances), DC, and mechanical properties of TPP printed materials. As writing power is increased and writing speed reduced, higher degrees of conversion are achieved, leading to enhanced material properties such as apparent elastic modulus for TPP printed structures.^[^ ^54^ ^]^ The consistent trends observed across all experiments strengthen confidence in both the model and the data.

#### 2.4.3 Hatching Angle, Slice Distance, and Fiber Size Effects

**Figure 5:**
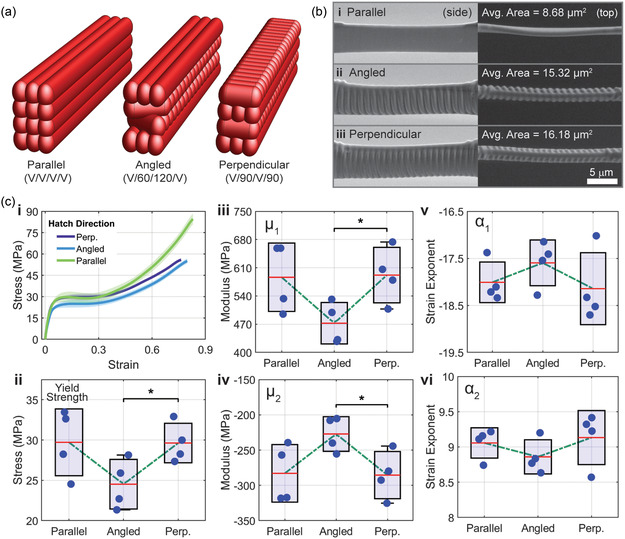
Evaluation of hatching style on fiber geometry and mechanical properties. a) Schematic of three hatching styles with layer descriptions of IP‐S: parallel (V/V/V/V), angled (V/60/120/V), and perpendicular (V/90/V/90), showing the stacking and alignment of fibers (parameter constants; LP: 30 mW, WS: 50 mm s^−1^, W × H: 1 × 2 μm, S × H: 0.5 × 0.5 μm). b) SEM images of fibers fabricated with i) parallel, ii) angled, and iii) perpendicular hatching styles, illustrating differences in geometry and surface texture from side and top views (scale bar = 5 μm). c) Comparison of stress–strain data and fit‐parameters for hatch styles of parallel, angled, and perpendicular (*n* = 4, 4, and 4). i) Representative engineering stress–strain curves; ii) yield stress $\sigma_{y}$; iii,iv) Ogden moduli, $\mu_{1}$and $\mu_{2}$; and v,vi) Ogden strain exponents, $\alpha_{1}$ and $\alpha_{2}$. In all panels, boxes represent mean ± standard deviation, whiskers indicate the minimum and maximum observed values, and dots are individual replicates.

The effect of layer‐to‐layer hatching angle—parallel, 0°, angled, 60°, and perpendicular, 90°—on fiber mechanical properties revealed subtle yet notable differences (**Figure** **5**). Consistent with findings from the fiber bridge study, angled and perpendicular‐hatched fibers exhibited increased sizes, likely due to line scan overshoot during fabrication. The angled fiber structure consisted of alternating layers: one vertical, followed by two layers angled at 60° and 120°, and then another vertical layer (V/60/120/V), as shown in Figure 5a. These fibers demonstrated reduced strength, which can be attributed to their discontinuous or ridged structure, as the increased area measurement does not fully account for these irregularities (Figure 5b). In contrast, the perpendicular‐hatched fibers, composed of alternating vertical and horizontal layers (V/H/V/H), maintained comparable strength to parallel‐hatched fibers despite their larger cross‐sectional area. Unlike angled fibers, the perpendicular‐hatched structure appeared more continuous and cohesive, though ridges were still visible (Figure 5b). Both angled and perpendicular‐hatched fibers exhibited distinct straining mechanisms, as observed during real‐time testing. Specifically, these fibers tended to “split,” or separate, between the fiber ridges produced from the horizontal components of their writing paths. Despite these observed differences, the mechanical performance of the fibers suggests relatively consistent laser energy delivery across their volumes. This uniform energy delivery minimizes significant anisotropic behavior, meaning the mechanical properties remain comparable as long as the energy density in fabrication of the fiber remains consistent. However, further reductions in laser power or decreased voxel overlap could enhance the effects of the writing path, potentially amplifying differences in fiber structure and performance.

Another separate study aimed to investigate the “meta‐structural” effects of slice and hatch distances on fiber mechanical behavior. Fibers fabricated with varying slice and hatch distances demonstrated relatively consistent mechanical properties, with the most significant differences arising in fiber size (Figure S5, Supporting Information). Decreasing hatching and slicing distances increased voxel overlap and energy delivery, resulting in pronounced radical diffusion (Figure S5b, Supporting Information). Similarly, as suggested before, lowering the laser power or increasing slice and hatch parameters in future studies may enhance the prevalence of metastructural effects, providing further insights into the tunability of mechanical properties.

#### 2.4.4 Material Comparisons

The TPP μTT demonstrates versatility in evaluating a wide range of photo‐initiated materials, as shown through a mechanical comparison of different resins. In addition to IP‐S, the resins IP‐Dip, IP‐Visio, and IP‐PDMS were tested. For multimaterial studies, IP‐S served as the base material for μTT structure fabrication. After fabrication, the structures were washed, and fibers composed of the alternative materials were deposited and printed on top of the base structures. To determine each resin's Young's modulus, the linear portion of each curve was fitted and its slope value obtained.

**Figure 6:**
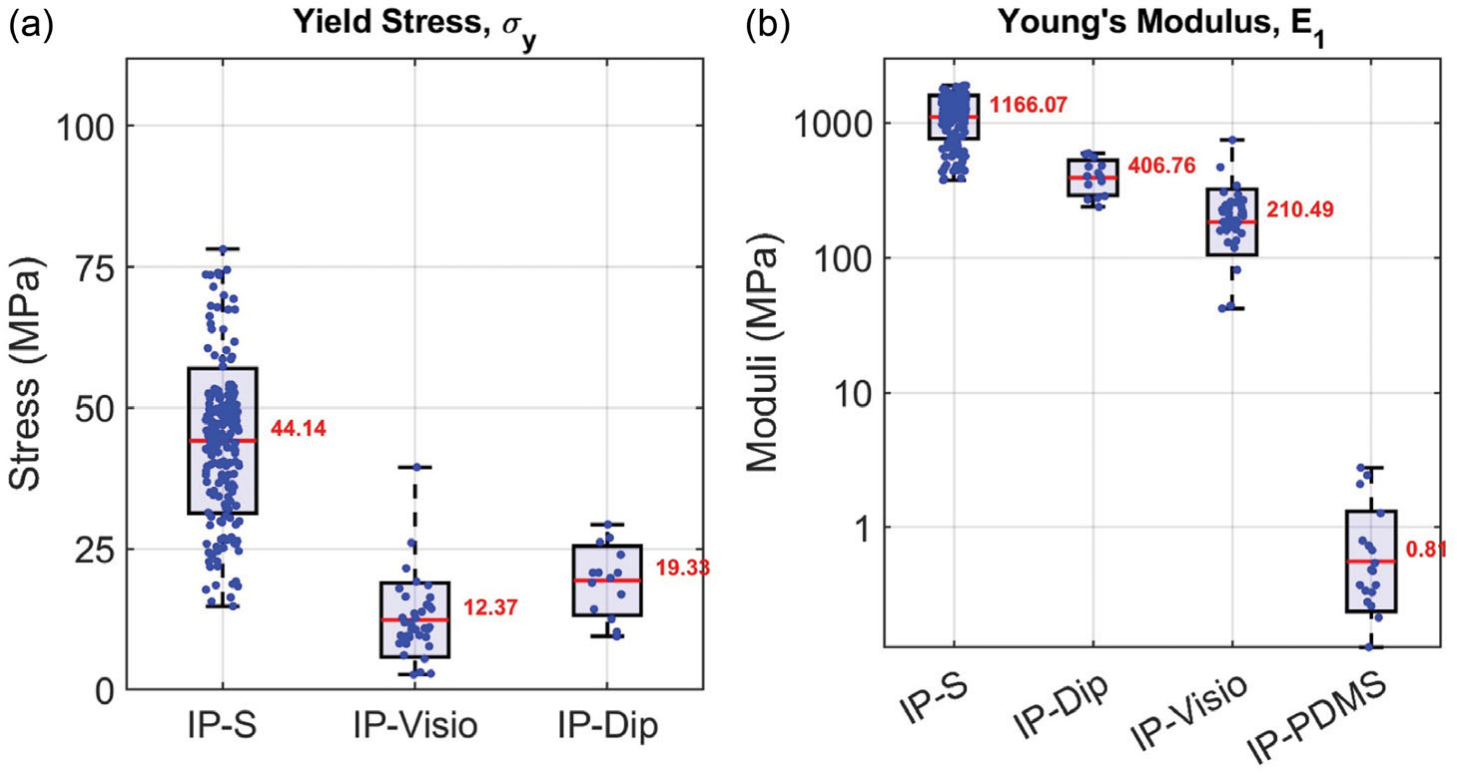
Mechanical property comparison of Nanoscribe resins. Comparison of three Nanoscribe resins (IP‐S, IP‐Dip, IP‐Visio, and IP‐PDMS (for Young's Modulus)) evaluated under submerged water conditions at ambient room temperature (*n* = 239, 14, 40, 18). a) Yield stress ($\sigma_{\gamma }$). b) Young's modulus (*E*). IP‐S shows the highest yield strength and stiffness, with IP‐Dip and IP‐Visio demonstrating progressively lower strengths and stiffness. IP‐PDMS exhibits a dramatically reduced Young's modulus and was not able to be tensioned to a yield strain. All data show statistical significance (*p* < 0.001). In all panels, boxes represent mean ± standard deviation, whiskers indicate the minimum and maximum observed values, and dots are individual replicates.

The results, summarized in **Figure** **6**, demonstrate the distinct mechanical behaviors of each material and the tunability of their properties through TPP processing. IP‐S exhibited the highest strength and stiffness, with an average yield stress of 44 MPa and a Young's modulus of 1166 MPa. Its moderate straining behavior positions it as a robust material for applications requiring strength and durability. IP‐Visio, by contrast, showed significantly lower strength (12 MPa yield stress) and stiffness (210 MPa Young's modulus) but displayed exceptional large strain deformation capabilities. Notably, IP‐Visio fully recovered from large deformations under ambient conditions, suggesting unique deformation recovery properties.^[^ ^64^ ^]^ IP‐Dip demonstrated intermediate properties, with strength (19 MPa Y.S.) and stiffness (406 MPa Y.M.) values higher than IP‐Visio but lower than IP‐S, and the most limited straining behavior. IP‐Dip is one of the most commonly tested IP‐resins and displays some of the most efficient and precise crosslinking; a literature review table of methods and measurements can be found in Table S1, Supporting Information. IP‐PDMS showed the lowest stiffness of all resins employed in this study (0.81 MPa Y.M.) which aligns with values ranging from 350 kPa to 17.8 MPa previously reported.^[^ ^59^ ^]^ Interestingly, IP‐PDMS samples could not be strained to an extent to induce the yielding of the fiber (shown from the stress–strain curve in Figure S9, Supporting Information), demonstrating its soft and highly flexible behavior even at strains considered large for other resins. The findings presented here highlight the tunability of photopolymerized resins and the critical influence of processing conditions on material behavior. All tests were conducted in submerged water at ambient room temperature, providing a unique and controlled environment for consistent comparisons.

Notably, both IP‐Visio and IP‐S's main component (>95%) is a diurethane dimethacrylate monomer (7,7,9‐trimethyl‐4,13‐dioxo‐3,14‐dioxa‐5,12‐diazahexadecane‐1,16‐diyl bismethacrylate). The difference in these materials underscore the role of crosslinking and photoinitiator composition in dictating mechanical performance and material behaviors. For instance, IP‐Visio demonstrates full shape recovery under submerged ambient conditions, but weaker mechanical properties. In contrast, the IP‐S polymer studied here showed only partial recovery and stronger mechanical properties. However, a heat‐assisted recovery cycle was effective in fully restoring the fiber to its original form, with slight changes in mechanical behavior (Figure S6, Supporting Information). Interestingly, both IP‐S and IP‐Visio required submersion in water to demonstrate full shape recovery.^[^ ^64^ ^]^ We hypothesize that this recovery depends on hydrogen interactions to increase molecular chain mobility to achieve recovery. These findings suggest that lower crosslinked densities within a polymer (IP‐Visio) enhance chain mobility and shape recovery, at the cost of mechanical strength.

#### 2.4.5 Cyclic Testing

**Figure 7:**
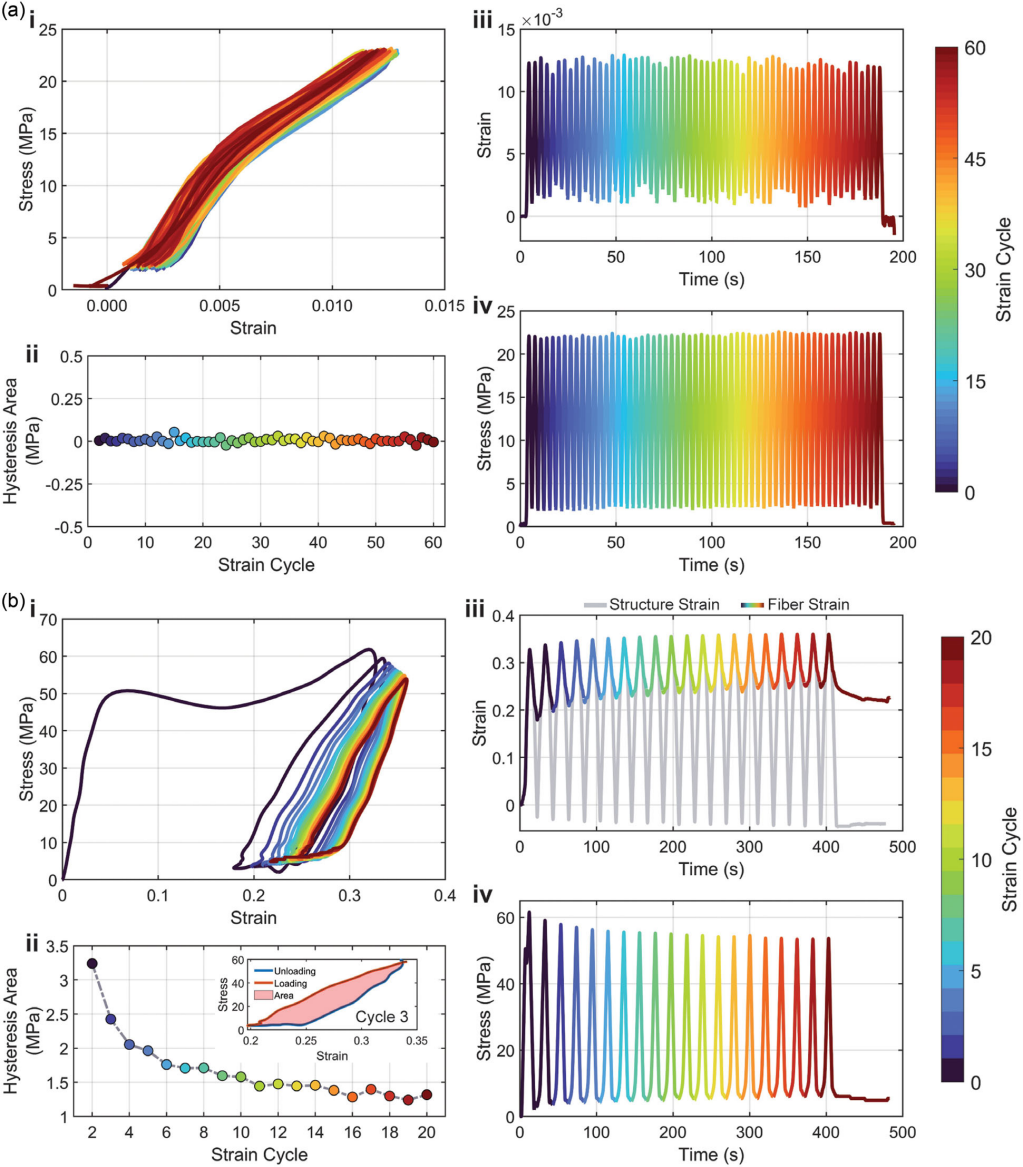
Cyclic testing of μTT structures of IP‐S under elastic and mid‐range strain conditions. a) Elastic strain cycling (≈1.25% max strain, 60 cycles. Fiber parameters: LP: 37.5 mW, WS: 55 mm s^−1^, W × H: 1 × 2 μm, S × H: 0.4 × 0.2 μm, Style: perpendicular, rate: 2 μm s^−1^). i) Stress‐strain curves. ii) Hysteresis area over strain cycles, confirming elastic behavior with no energy dissipation. iii) Strain versus time and (iv) stress versus time with cycles color‐mapped by strain cycle number. b) Mid‐range strain cycling (≈35% max strain, 20 cycles. Fiber parameters; LP: 30 mW, WS: 55 mm s^−1^, W × H: 1 × 2 μm, S × H: 0.4 × 0.2 μm, style: perpendicular, rate: 2 μm s^−1^): i) Stress–strain curves for larger strain cycles, demonstrating clear hysteresis. ii) Hysteresis area over strain cycles, showing decay with repeated cycling. Inset shows graphical hysteresis calculation method. iii) Structure and fiber strain versus. time. The graph shows contrasting curves of the fiber measured strain and structure measured strain, implicating fiber deformation, and iv) stress versus time.

To evaluate the potential for cyclic testing using the device, a variety of straining ranges and number of testing cycles were evaluated. **Figure** **7** shows two cases, an elastic straining experiment (≈1.25% max strain, 60 cycles) and a mid‐range strain experiment exceeding the threshold for plastic deformation (≈35% max strain, 20 cycles). Under elastic strain cycling, stress–strain curves exhibited negligible hysteresis, with hysteresis area values near zero across all cycles (Figure 7a(ii)), confirming purely elastic behavior. The consistent strain and stress amplitudes over 60 cycles indicated stable and repeatable cyclic performance, making the material well‐suited for high‐cycle durability applications requiring minimal energy dissipation (Figure 7a). In contrast, mid‐range strain cycling revealed viscoelastic behavior, characterized by pronounced hysteresis loops and measurable energy dissipation. The hysteresis area decreased exponentially over the first 10–15 cycles before stabilizing, suggesting strain‐induced relaxation and material stabilization (Figure 7b). In Figure 7b(iii), the straining is shown for both the fiber‐measured strain and the structure‐measured strain. The structure strain evaluates the forcing structure displacement in relation to the sensing structure displacement. The fiber strain is a pure measurement of the fiber length throughout the experiment. The difference between these measurements indicates fiber deformation, which is recorded through fiber buckling.

The TPP μTT device demonstrates exceptional versatility as a microscale tensile testing method, capable of assessing both elastic and viscoelastic material behaviors under cyclic loading. Future experiments could explore the limits of cyclic loading leading to fracture, expanding the understanding of fatigue life in microstructures. Furthermore, enhanced data analysis to determine the storage and loss behavior of these viscoelastic materials is underway. In large‐strain experiments, mitigating deformation in the μTT sensing structure by increasing beam thickness could enhance performance, ensuring the sensing beams remain within their elastic strain range and retain mechanical integrity. These insights highlight the adaptability and potential of the TPP μTT device as a robust platform for a wide range of high‐precision mechanical characterization methods.

## 3 Outlook

The TPP μTT device and process are well‐positioned to serve as a standardized method for microscale tensile testing, with the potential to address the growing need for reliable material characterization at the microscale. This approach facilitates the systematic evaluation of key mechanical properties, including Young's modulus and yield strength under biologically relevant conditions, such as submerged liquid environments. These conditions are particularly important for hydroresponsive TPP materials, which often swell and exhibit dynamic responses to physiological settings. We demonstrated that standardized protocols can be developed through systematic parameter variation, such as adjustments to laser power, writing speed, and hatching parameters, optimizing fiber formation and mechanical performance. The integration of automation holds a significant promise for advancing the μTT process, increasing throughput and enabling high‐efficiency testing. With automated positioning systems, TPP μTT arrays could be efficiently scanned and aligned for testing, streamlining data collection and processing. The μTT process has the capacity to accommodate large‐scale experiments, involving hundreds to thousands of tests per material, supporting robust statistical analysis and validation. When combined with complementary techniques, such as nanoindentation and micro compression, the μTT approach could offer a comprehensive evaluation of material behavior.

Grayscale TPP, in which laser power, and thus voxel size, is dynamically controlled based on a grayscale image of a desired geometry, shows great promise for improved print resolution, specifically in the field of micro‐optics.^[^ ^72^ ^]^ As seen in the presented study, laser power and resulting voxel dimensions greatly impact mechanical properties of printed structures. While the fibers tested in the scope of this research utilize consistent voxel size for individual prints, the μTT device could be employed for investigation of this new, higher resolution method of TPP, providing better understanding of the effect variable voxel size has on the cumulative material properties of a printed structure. Furthermore, the μTT device is flexible in its ability to accommodate the investigation of TPP metamaterials in future studies. Because the sensing structure of the device is additionally manufactured via TPP, its stiffness is easily adjustable via dimensional or printing parameter changes. Alignment and additional printing on top of the forcing and sensing structures have been demonstrated in this study, displaying the capability of the device in the potential printing of 3D TPP metamaterials in place of the fibers explored here. Metamaterial experiments have been explored previously but have yet to be fully investigated.^[^ ^64^ ^]^ Both grayscale TPP structures and metamaterials offer new and natural progressions for material characterization in the context of TPP.

Beyond immediate testing capabilities, the development of a TPP μTT material database presents an opportunity to facilitate material design through the integration of experimental data, molecular modeling, and machine learning.^[^ ^73^ ^]^ By correlating mechanical properties with fabrication parameters and material composition, such a database could serve as a resource for leveraging machine learning to design new materials with tailored mechanical and biological properties.^[^ ^74^ ^]^ Simultaneously, the integration of multiscale molecular modeling into TPP offers transformative potential for understanding and designing advanced microscale polymers. By simulating the crosslinking process at the molecular level, the intricate balance of physical and chemical interactions that govern polymer behavior can be studied. Molecular modeling enables the optimization of fabrication parameters, such as laser dosage, to precisely control geometry and fine‐tune mechanical properties.

In sum, the TPP μTT device serves as a critical tool for translating material research into impactful biomedical applications. By enabling precise mechanical characterization of TPP‐fabricated scaffolds, the μTT device ensures that mechanical properties such as stiffness, elasticity, and durability can be tuned to meet biological requirements. Furthermore, the μTT device facilitates testing under biologically relevant conditions, providing insights into scaffold behavior in submerged and dynamic environments. Its ability to evaluate various materials expands opportunities for integrating diverse biomaterials, offering a pathway to optimize designs for various tissue engineering and regenerative medicine applications. As a result, the TPP μTT device bridges the gap between material innovation and biological functionality, supporting the creation of complex, scalable, and clinically relevant tissue engineering solutions.

## 4 Experimental Section

4.1

4.1.1

##### TPP Fabrication

TPP was performed using a Nanoscribe Photonic Professional GT system. The fabrication process was designed and controlled using Nanoscribe's proprietary slicing software, Describe. A Dip‐in‐Laser Lithography (DiLL) configuration with a 25× objective was utilized for all experiments. The substrates used were 18 mm circular glass that had been plasma‐treated to apply an indium tin‐oxide (ITO) surface coating. The ITO layer had a thickness of ≈50 nm and was deposited using direct current sputtering of an ITO target in an Ar plasma. An additional layer of porous silicon oxide (PSO), with a thickness ≈2 μm, was deposited on top of the ITO providing a high density of nanopores providing surface anchoring of TPP printed materials. Each substrate was secured to the TPP printer's substrate holder using adhesive tape. The resin was deposited on the central portion of the substrate prior to loading. Substrates were mounted in an inverted orientation, allowing the microscope objective to be dipped directly into the resin (Dip‐in‐Laser configuration). The surface was brought into focus before the printing process commenced.

Nanoscribe resins—IP‐S, IP‐Dip, IP‐Visio, and IP‐PDMS—were employed in this study. For the primary μTT device fabrication, IP‐S resin was used exclusively. Consistent printing parameters were maintained at 60% laser power and a scanning speed of 55 mm s^−1^ to ensure reproducibility. After printing, the substrate was removed from the holder and immersed in SU‐8 developer (1‐methoxy‐2‐propyl acetate) to dissolve unpolymerized resin. Structures were soaked in the developer for at least 1 h, then washed with isopropyl alcohol (IPA) and deionized water. The structures were subsequently dried using a gentle stream of filtered nitrogen and packaged for shipment.

For experiments requiring materials other than IP‐S, a two‐step fabrication process was employed. In the first step, structural arrays were printed using IP‐S resin as the base material. After development and drying, a small amount (≈0.1 mL) of the second material resin (e.g., IP‐Dip, IP‐Visio, or IP‐PDMS) was applied to the substrate. The substrate was then reloaded into the TPP printer, and the microscope objective was focused within the new resin layer. The IP‐S printed structures were located and aligned. Fiber elements made from the second resin were subsequently printed on top of the existing structures. The resins used in these multimaterial experiments were photocompatible, enabling effective bonding between the layers. This approach demonstrated strong adhesion and mechanical integration between the materials. In total, production of arrays consisting of both devices and their fibers took ≈8 h for IP‐S fibers, and 10 h for arrays requiring fibers of different resins.

##### Bridge Study

The IP‐S fiber‐bridge arrays were printed with six different beam specifications, and each structure contained 15 bridges ranging from 20 to 90% (10–45 mW) power. Then, for each specification, there were 6 writing speeds evaluated (20–70 mm s^−1^). This totaled 90 fibers per beam specification and 540 fibers for the study. The fiber bridge arrays were analyzed using the Zeiss Merlin SEM at ORNL. This SEM did not require coating of the samples. A custom MATLAB algorithm was used to analyze the fiber geometry. SEM data collection takes ≈8 h to complete.

##### TPP μTT Testing

The TPP μTT arrays were adhered to a low‐edge glass bottom substrate (PELCO Clear Wall Glass Bottom Dishes) using an optically transparent and fast‐curing adhesive (Norland Optical Adhesive 86H). A small volume of adhesive was placed in the center of the dish, and the substrate was gently placed on top. The substrate was exposed to UV light (12 W, 365 nm) for 5 s to cure the adhesive. The substrate was then washed with distilled water until the structures did not have bubbles trapped around them. Then, a final washing was conducted, and the substrates were submerged in water for at least 2 h. The substrates were then prepared for testing.

Testing was conducted with a Nanosurf AFM system with a C3000 controller. The system was mounted on an inverted Zeiss Microscope. The AFM probe used to actuate the forcing structure was a modified tipless cantilever (TL‐NCL, Nanosensors). The probes were modified with FIB (FEI Helios FIB/SEM 660), where the tips were removed and a 13 μm hole was milled for hooking the pillar of the forcing structure. The structures were loaded onto the microscope with tedious alignment to ensure verticality in testing. Then, the AFM probe was moved into position and hooked to the pillar. The displacement rate and magnitude were selected, and a maximum strain holding time was determined. Screen recording software (Camtasia) was used to capture videos of the testing. Tests were then conducted on the μTT array. The average tensile testing time is 3 min, and 1 additional minute is required for movement between samples. A full array of μTT devices takes ≈6 h to fully test.

##### TPP μTT Analysis

Data postprocessing began by converting raw experimental data into empirical data through a series of transformations. These included applying a pixel‐to‐micron conversion, calculating the force on the sensing structure, determining strain, and dividing the force by the cross‐sectional area to compute stress (see Supplementary Section 1, Supporting Information). The pixel‐to‐micron ratio, determined from initial experiments using known feature sizes and experimental distances, was calculated from raw video images. Experiments conducted at 10× magnification established a standard conversion ratio of 0.33 microns pixel^−1^. A custom, in‐house digital image analysis (DIA) algorithm was employed to process experimental data (Supplementary Section 2, Supporting Information). This algorithm utilized image enhancement (4× pixels, yielding 0.0825 microns pixel resolution^−1^), binarization, bulk grid matching, and edge analysis to achieve sub‐pixel resolution displacement tracking. The DIA algorithm processed experiments at ≈1 frame per second on a Dell Precision 5810 workstation, with two MATLAB instances running simultaneous evaluations. The custom MATLAB scripts used for this study's analysis can be found on the lab's GitHub page (github.com/YangLabMSU/MicroTT). Raw data, captured at 10 frames per second, typically comprised of ≈1000 frames for a standard test cycle of 40 μm displacement, 60 s hold, and return, resulting in a total test duration of ≈100 s. With other, longer experiments accounted for, the average test time is 3 min. The DIA script operates at 1 s per frame, with recordings containing, on average, 2700 frames. Each video analysis takes roughly 45 min. Full MATLAB analysis of a complete array (80 devices) would take 60 h total to complete. Together, all data collection and analysis of a full array takes 74 h.

##### Statistical Analysis

Box plots were employed to visually represent the data distribution for each group, highlighting key statistical metrics. Sample sizes are included within each figure caption. In each box plot, the central line indicates the mean value, while the box boundaries represent the standard deviation range. Whiskers extend to the minimum and maximum values within the data, excluding outliers. Individual data points are overlaid as scatter plots to illustrate variability within the groups. Statistical comparisons were performed using two‐sample *t*‐tests to evaluate differences between experimental groups. Significance levels were denoted on the plots using brackets and asterisks (* for *p* < 0.05, ** for *p* < 0.01, and *** for *p* < 0.001), with statistical annotations placed above the corresponding categories. If all samples differed significantly, the plot annotations were omitted, and significance was stated in the figure's caption. All statistical analyses and visualizations were performed in MATLAB using a custom script designed to ensure reproducibility and accuracy.

## Conflict of Interest

The authors declare no conflict of interest.

## Supporting information

Supplementary Material

## Data Availability

The data that support the findings of this study are available in the supplementary material of this article.
