# Supplementary material for: Toward Standardized Microscale Tensile Testing for Two‐Photon Polymerization‐Fabricated Materials in Liquid
Source: Small Sci. 2025 Jul 22;5(9):2500228. doi: 10.1002/smsc.202500228 (PMC12412484; doi:10.1002/smsc.202500228)
Supplement: Supplementary file 1 — Supplementary Material [file SMSC-5-2500228-s001.pdf]

Supplementary Information

Towards a Standardized Microscale Tensile Testing of TPP Fabricated Scaffolds in Liquid

Grayson Minnick, Timothy Goldsmith, Bahareh Tajvidi Safa, Amir Ostadi Moghaddam, Jordan Rosenbohm, Nickolay V. Lavrik, Wei Gao, Ruiguo Yang**

Supplementary Contents

1. Data Processing 2
2. Digital Image Analysis for TPP µTT Experiments 3
3. SEM Evaluation of Power and Write Speed Samples 6
4. Hatch and Slice Distance Evaluation 7
5. Heat Recovery and Fatigue Evaluation 9
6. Additional Fiber Bridge Data 10
7. IP-PDMS Stress-Strain Curve Comparison 12
8. Literature Review of IP-Dip 13
References 14

Supplementary Figures

Figure S1. Algorithm Flow
Figure S2. Analysis Summary and Small Deformation Sensitivity
Figure S3. SEM Measurements of Fiber Area for Writing Power Data
Figure S4. SEM Measurements of Fiber Area for Write Speed Data
Figure S5. Impact of Slice and Hatch Distances on Fiber Geometry and Mechanical Properties
Figure S6. Fatigue Testing and Heat Recovery in TPP µTT Structures
Figure S7. Angled Fiber Geometry
Figure S8. Perpendicular Fiber Geometry
Figure S9. IP-PDMS Stress-Strain Curve Comparison

Supplementary Tables

Table S1. IP-Dip Modulus Characterization from previous literature.

1. Data Processing

The raw experimental data was first loaded and converted into measurable parameters using the micron-per-pixel ratio (MPR). This conversion ensured that all data was expressed in terms of top structure displacement, d, bottom structure deformation, δ, and fiber length, L. These parameters formed the basis for calculating force, stress, and strain during the experiments.

The known and calibrated beam stiffness (k_{sens}) was used to convert the deformation of the sensing structure into force (F_{sens}). Following this, engineering stress (σ_{fiber}) was determined by dividing the force by the determined area of the fiber (A_{fiber}).

$$F_{sens} = k_{sens} \cdot \delta_{sensing} \quad (1)$$

$$\sigma_{fiber} = F_{sens}/A_{fiber} \quad (2)$$

Strain was determined using the initial length of the fiber ($L_{o,fiber}$), and was calculated in two forms. The first measurement was coined structure strain, $\varepsilon_{structure}$. The structure strain used relative displacements of the forcing ($d_{actuation}$) and sensing ($\delta_{sensing}$) structures to determine the strain. This strain calculation method was accurate for the stretching and holding portion of the experiments, but it was inaccurate when returning the structures to their initial position, where the fiber was deformed or buckled. The fiber strain, (ε_{fiber}), used the fiber measurement to determine the strain of the fiber. This measurement was able to accurately capture the length of the fiber when it was buckled.

$$\varepsilon_{structure} = \frac{d_{actuation} - \delta_{sensing}}{L_{o,fiber}} \quad (3)$$

$$\varepsilon_{fiber} = \frac{L_{fiber} - L_{o,fiber}}{L_{o,fiber}} \quad (4)$$

Following these conversions, the data underwent a smoothing process to reduce noise while retaining the integrity of the measurements. Smoothed data were plotted alongside the raw data for comparison, and each experiment was individually assessed to ensure the accuracy and reliability of the processed results.

2. Digital Image Analysis for TPP µTT Experiments

The development of a robust digital image analysis algorithm was critical for enabling high-throughput tensile testing of TPP µTT structures. The algorithm evolved from an initial pixel-by-pixel digital image correlation (DIC) method that, while achieving sub-pixel resolution, was hindered by pixel drift and high computational demands. To overcome and alleviate these limitations, a multi-step process was developed, incorporating bulk grid matching, edge detection and adjustment, and fiber length measurement techniques.

The analysis begins with video frame interpolation to enhance resolution, followed by advanced binarization methods to clearly define grid regions for the top, bottom, and fiber structures. Bulk grid matching identifies displacement by matching binarized grids across successive frames, significantly increasing efficiency and eliminating drift errors present in pixel-by-pixel methods. While effective for experiments involving large displacements, bulk grid matching can produce rough data under suboptimal video conditions and struggles to fully capture minimal displacements in weaker fibers. To address these issues, an edge adjustment algorithm was introduced. This algorithm calculates average relative positions of the structure edges to the bulk grid, enabling precise sub-pixel displacement resolution. The final step, fiber length measurement, utilized edge detection combined with Fourier model fitting to calculate the fiber's arc length through deformation. This approach provided accurate strain measurements, which were essential for analyzing cyclical and recovery testing. High-throughput batch analysis was achieved by automating the binarization and processing of entire arrays of tensile structures, with real-time plots offering immediate feedback on data quality and reliability. This streamlined workflow ensured efficient evaluation of large datasets, reducing processing times and enabling robust statistical analysis. This optimized algorithm represents a cornerstone for high-throughput microscale tensile testing of TPP-fabricated structures. It not only enables accurate and efficient data processing but also paves the way for future automation, further enhancing its potential for large-scale applications in material testing and characterization.

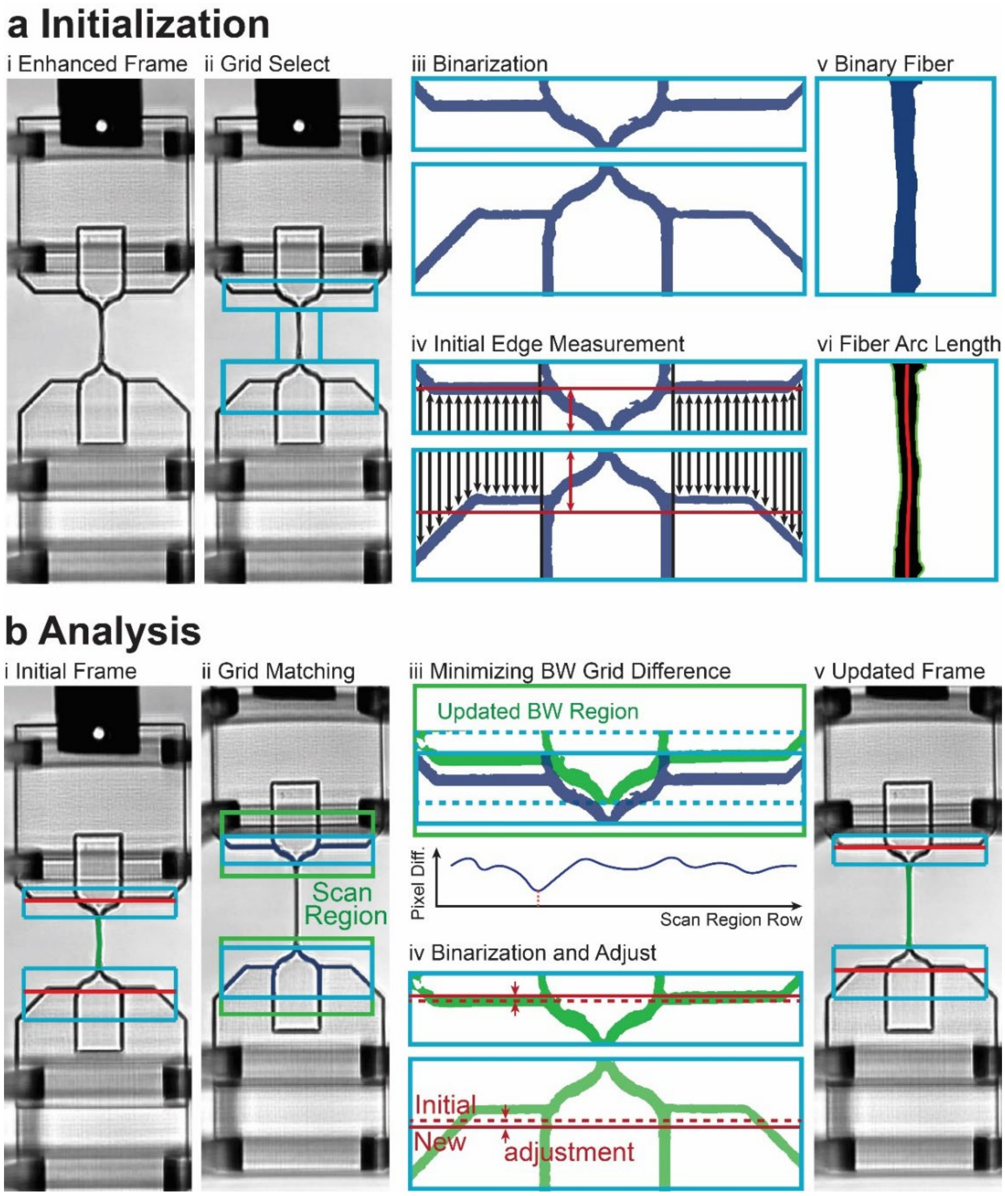

Figure S1. Algorithm Flow. a) First frame analysis and initialization. (i) Frame and enhancement (4x pixel enhancement), (ii) grid selection, (iii) grid binarization, (iv) initial mean edge location for displacement adjustment, (v & vi) binarized fiber and arc length measurement – this process is consistent for analysis of all frames. b) Displacement analysis flow. (i) Initial frame for comparison, (ii) initial frame, top and bottom black and white (BW) grids are scanned around the estimated region of deformation for difference minimization and grid matching. (iii) Visualization of grid minimization. The initial grid is compared to the updated grid. The minimized region of pixel difference is the matched grid displacement. The algorithm compares the initial and previous grid and specifies a threshold for accurate matching. (iv) Fine grid adjustment is done by finding the mean edge location and comparing it to the relative initial location, (v) the frame updates and moves to the next frame.

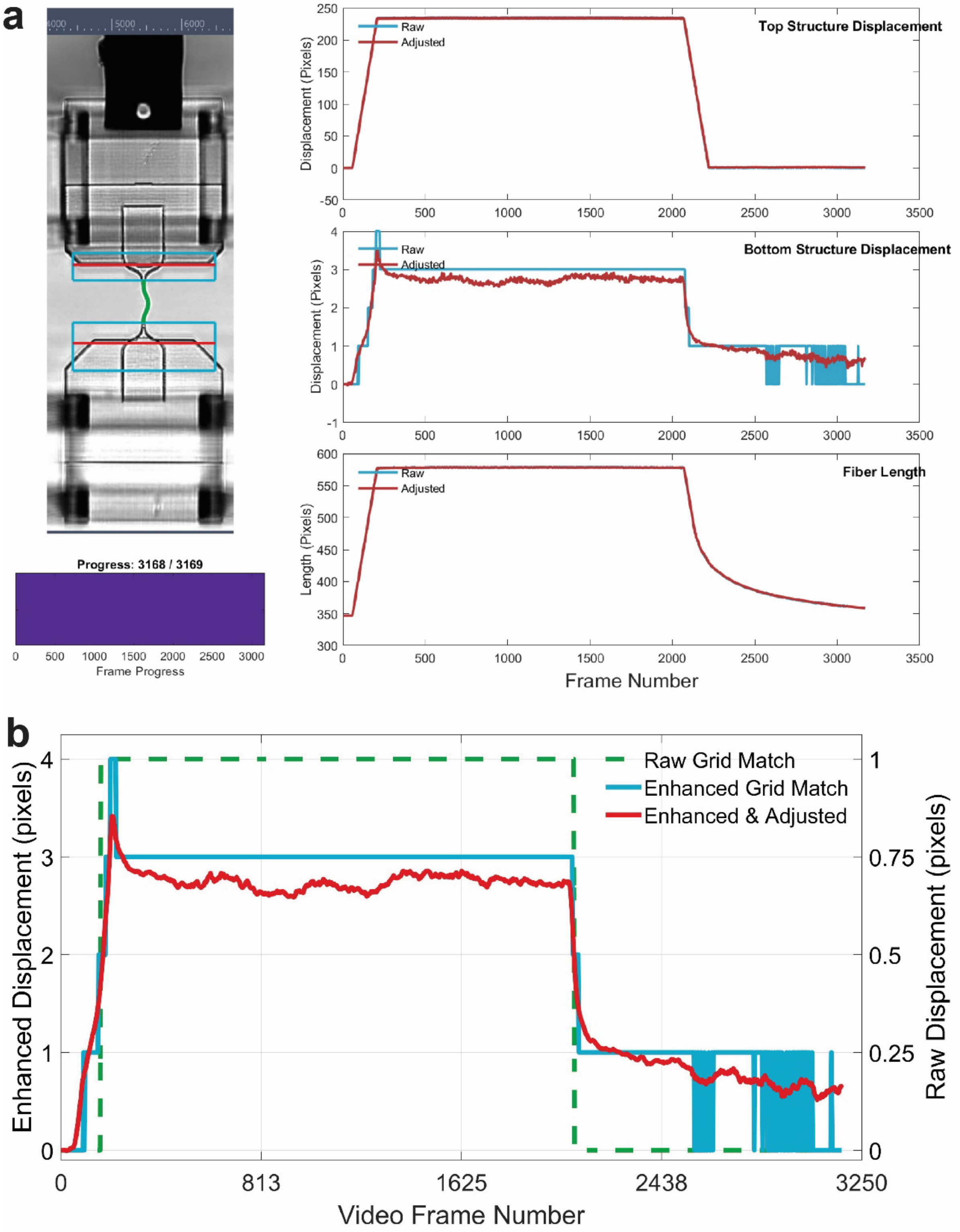

Figure S2. Analysis Summary and Small Deformation Sensitivity. a) Each analysis outputs the summary, which is easily verified upon analysis of experimental batch. The summary shows the final frame, and the raw analysis of the top displacement, bottom displacement, and fiber length. b) The algorithm has a sub-pixel resolution capability as demonstrated by the plot. Here, the different methods of analysis are compared, including raw grid matching, enhanced frame grid matching, and enhanced and adjusted analysis.

3. SEM Evaluation of Power and Write Speed Samples

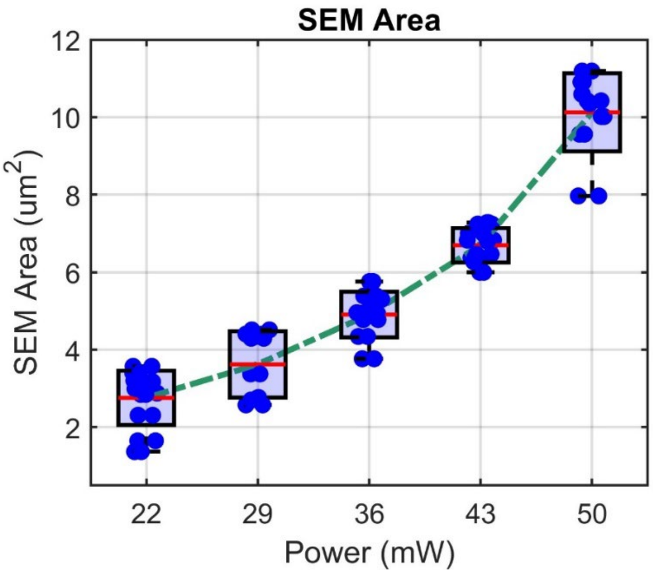

Figure S3. SEM Fiber Area vs. Laser Power. Fiber measurements for writing power data that is presented in Figure 4a.

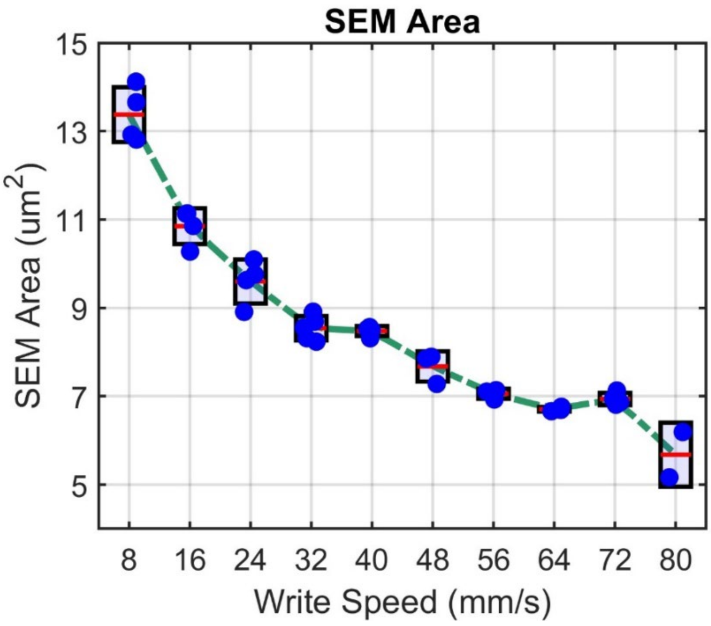

Figure S4. SEM Fiber Area vs. Writing Speed. Fiber measurements for writing speed data that is presented in Figure 4b.

4. Hatch and Slice Distance Evaluation

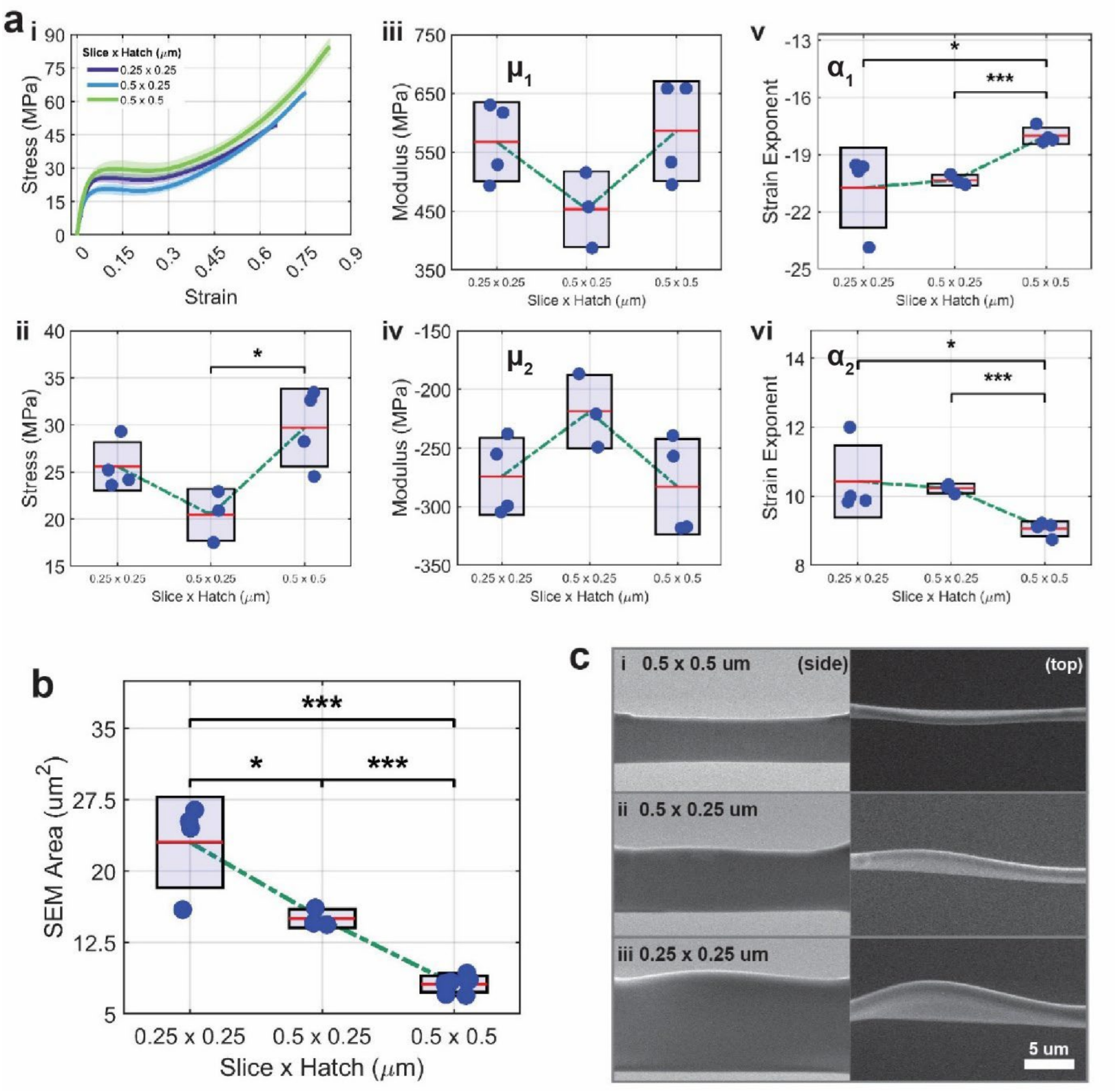

Figure S5. Impact of Slice and Hatch Distances on Fiber Geometry and Mechanical Properties. a) i: Comparison of stress-strain data and fit-parameters for fibers fabricated with different slice (S) and hatch (H) distances: 0.5 × 0.5 μm, 0.5 × 0.25 μm, and 0.25 × 0.25 μm (n = 4, 3, and 4. Constant Parameters; P: 30 mW, WS: 50 mm/s, W x H: 1 x 2 μm, Vertical). (i) Representative engineering stress–strain curves, (ii) yield stress σ_y, (iii & iv) Ogden moduli, μ_1 and μ_2, and (v and vi) the Ogden strain exponents, α_1 and α_2. In all panels, boxes represent mean ± standard deviation, whiskers indicate the minimum and maximum observed values, and dots are individual replicates. b) SEM fiber measurements (n = 4, 3, 5) and c) representative SEM images, highlighting differences in geometry, with side and top views shown. The SEM areas of the fibers show increasing size with reduced slice and hatch distances, correlating to greater overlap, leading to increased energy delivery and increased radical diffusion during fabrication. These results demonstrate that slice and hatch distances play a critical role in influencing fiber geometry. The increased voxel overlap leads to excessive energy delivery, which promotes radical diffusion and results in the expansion of fiber structure geometry. This radical diffusion likely reflects the upper threshold of crosslink conversion within the focal region. Consequently, the power and writing speed parameters used in this study may not fully capture the effects of hatch and slice manipulation.

Future studies could focus on exploring the lower threshold of crosslinking to further elucidate and amplify the influence of hatching and slicing parameters on fiber geometry and mechanical properties.

5. Heat Recovery and Fatigue Evaluation

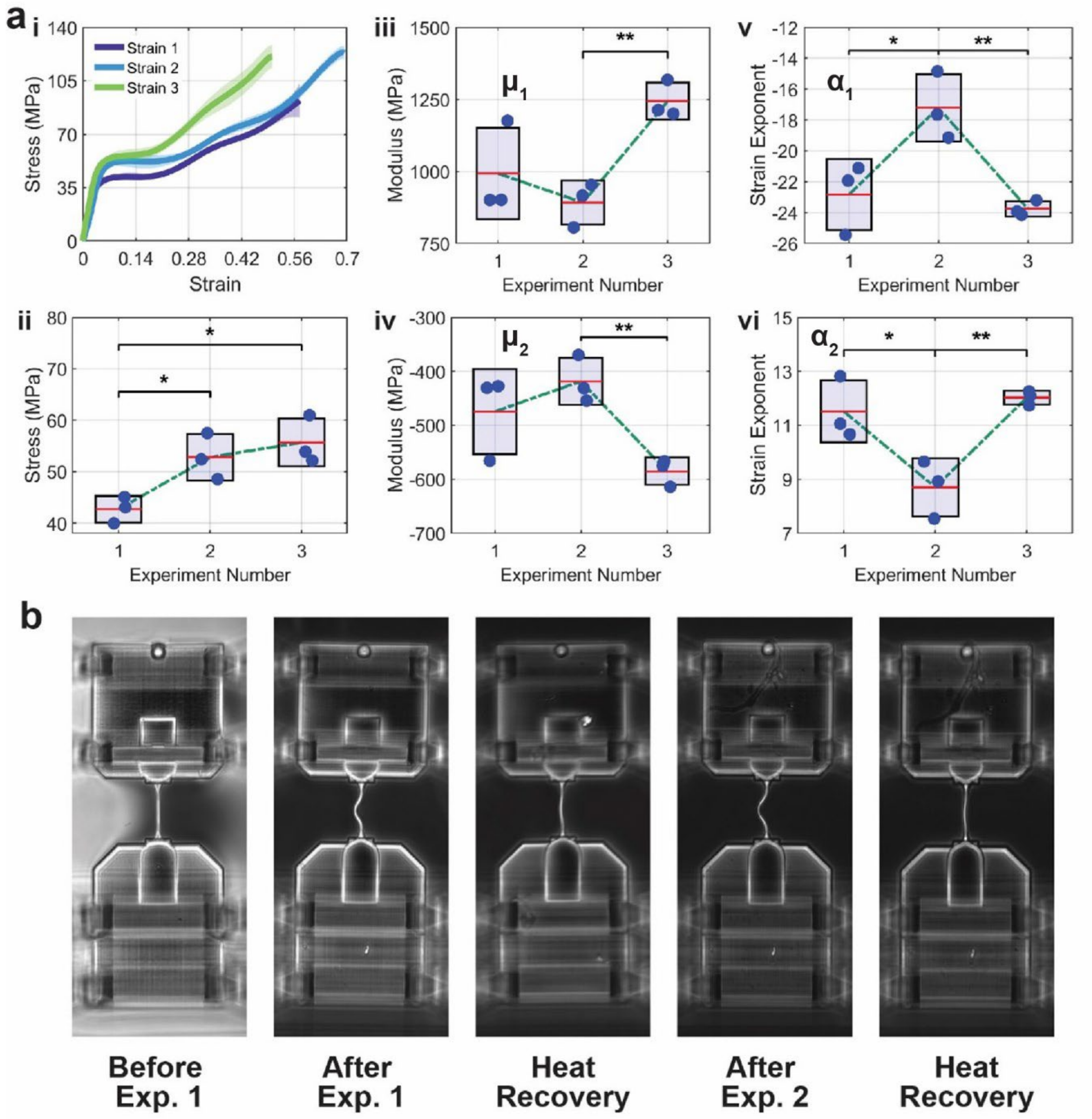

Figure S6. Fatigue Testing and Heat Recovery in TPP μTT Structures. a) Evaluating the effects of heat recovery on fiber mechanics (LP: 36 mW, WS: 55 mm/s, W×H: 1x2 μm, S×H: 0.4×0.2 μm, HS: Cross). (i) Representative stress-strain curves for a single fiber in multiple experiments after heat recovery, (ii) yield stress σ_y, (iii & iv) Ogden moduli, μ_1 and μ_2, and (v and vi) the Ogden strain exponents, α_1 and α_2. In all panels, boxes represent mean ± standard deviation, whiskers indicate the minimum and maximum observed values, and dots are individual replicates. b) Optical images of TPP μTT structures before and after strain experiments and subsequent heat recovery cycles. Images show deformation after each strain event and recovery following heat application. From the limited results of this study, it appears that there is a gradual increase in yield strength with successive experiments after recovery. The Ogden parameters also show a general trend of increasing magnitude for moduli, and relative stability in the strain exponents, though experiment two does not fit within this trend. Here, heat affects polymers primarily by increasing molecular mobility, indicating that the ambient conditions of testing may be near the glass transition temperature of IP-S.

6. Additional Fiber Bridge Data

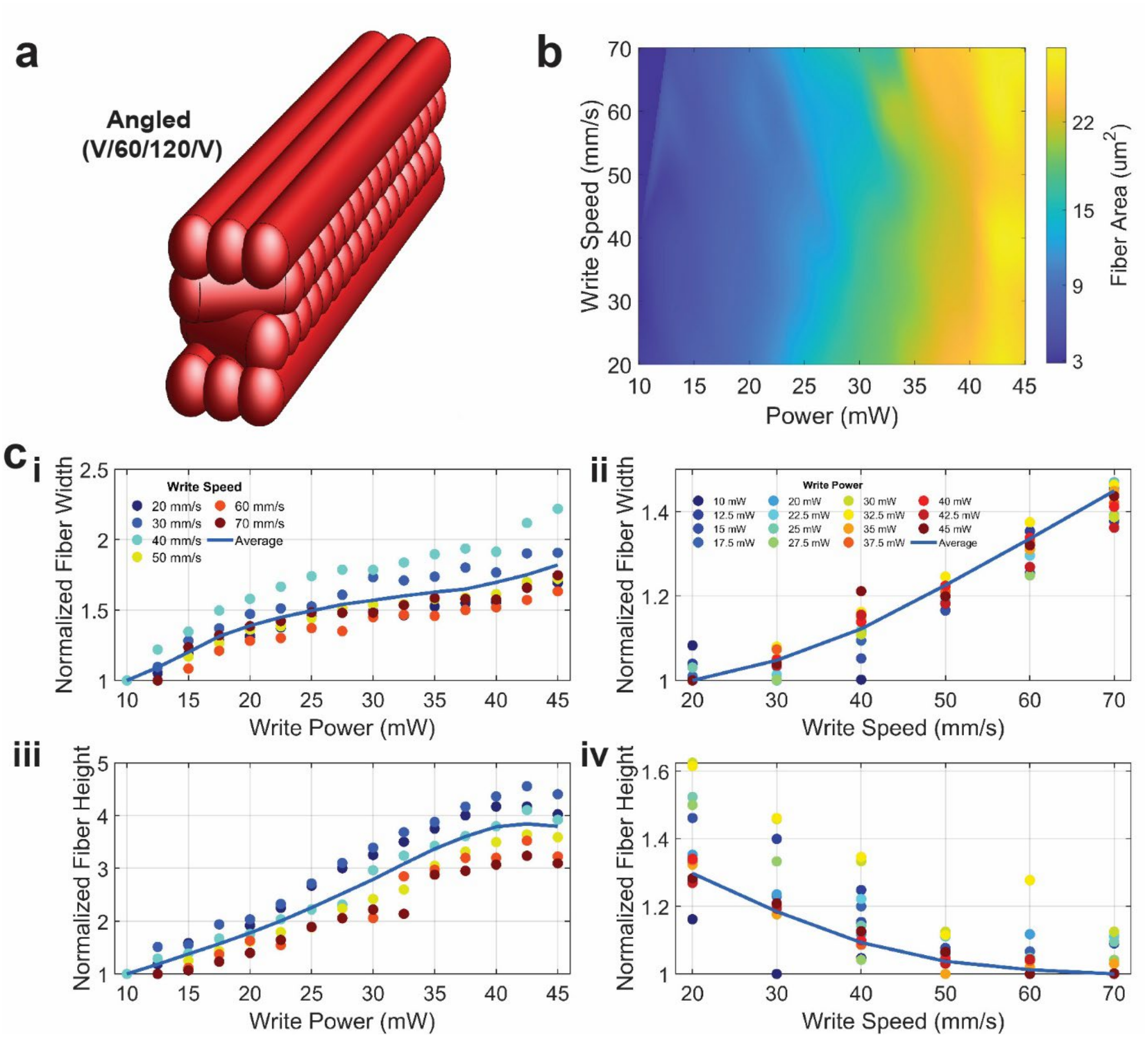

Figure S7. Angled Fiber Geometry. a) Angled fiber line writing illustration. b) Area heat map of writing speed vs. writing power. c) Normalized fiber measurements, (i) normalized fiber width vs. write power, (ii) normalized fiber width vs. write speed, (iii) normalized fiber height vs. write power, (iv) normalized fiber height vs. write speed.

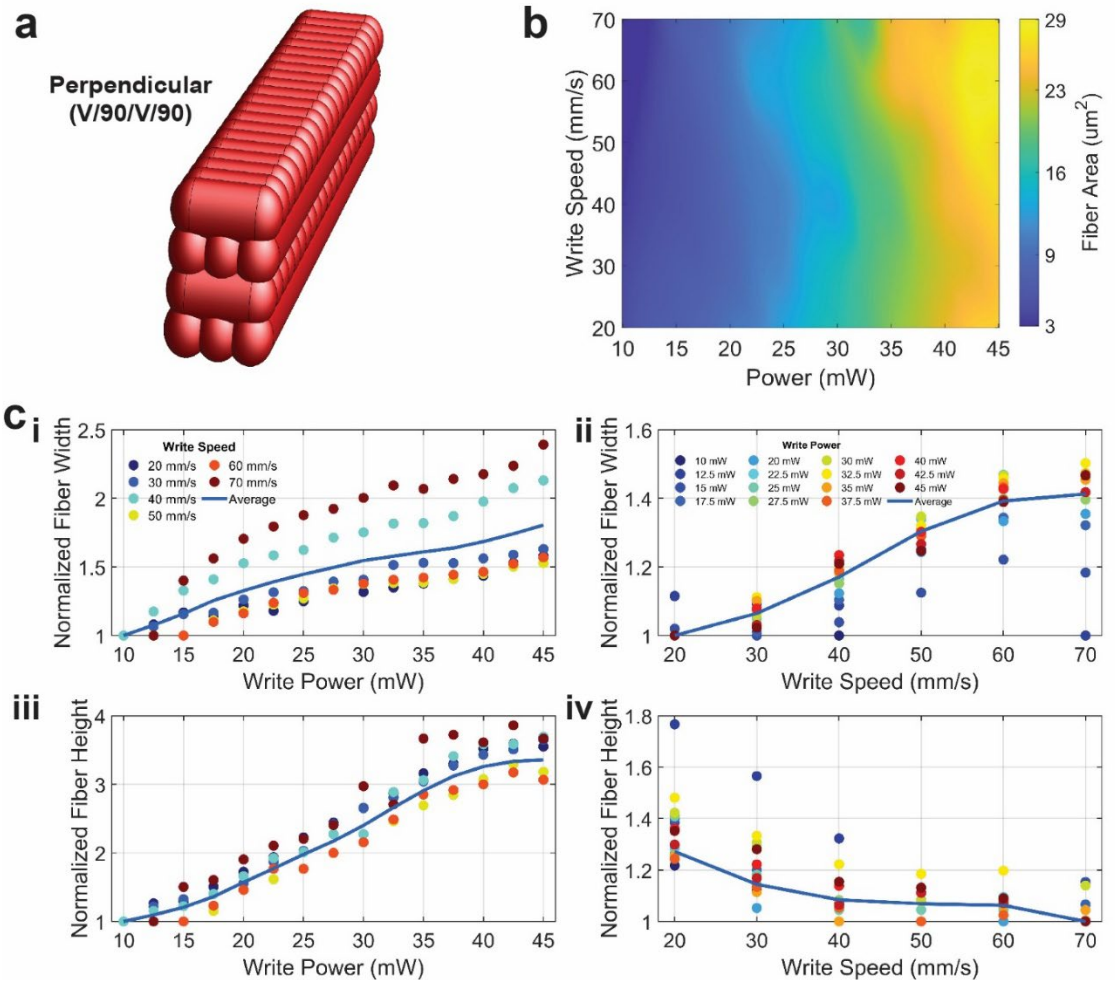

Figure S8. Perpendicular Fiber Geometry. a) Perpendicular fiber line writing illustration. b) Area heat map of writing speed vs. writing power. c) Normalized fiber measurements, (i) normalized fiber width vs. write power, (ii) normalized fiber width vs. write speed, (iii) normalized fiber height vs. write power, (iv) normalized fiber height vs. write speed. Here it is shown, that against intuition of laser exposure, that with increasing writing speed, fiber width tends to increase for both angled and perpendicular configurations. This is caused by overshooting the laser during galvo scanning of the angled and perpendicular lines with respect to the vertical.

7. IP-PDMS Stress-Strain Curve Comparison

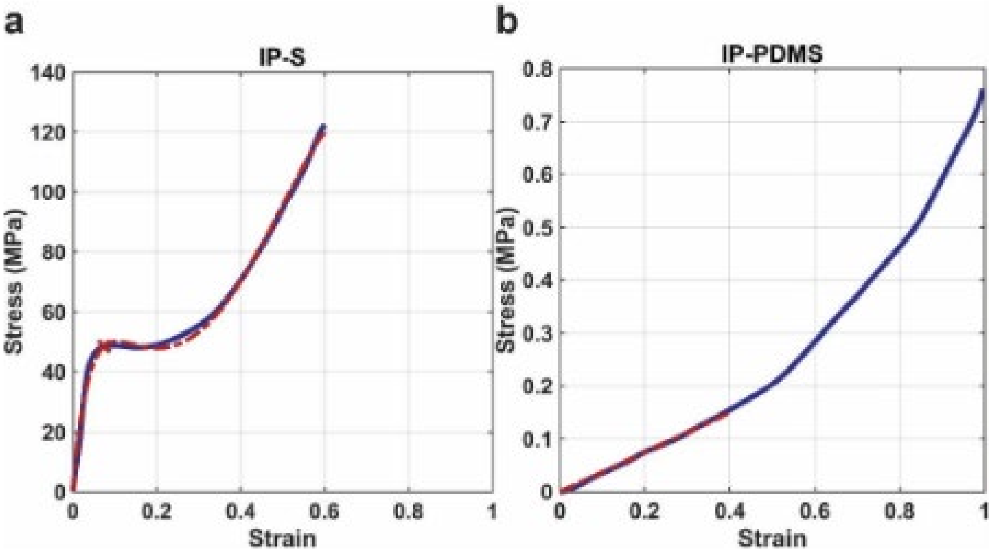

Figure S9. IP-PDMS Stress-Strain Curve Comparison. a) An exemplary stress strain curve of an IP-S fiber tensile test. The blue solid line is the smoothed data obtained from the experiment. The red dashed line shows the Ogden hyperelastic model fit to the curve. The red X indicates the yield stress of the fiber. b) An exemplary stress-strain curve of an IP-PDMS fiber tensile test. The blue solid line is the smoothed data obtained from the experiment. The red dashed line shows a linear fitting up to a strain of 0.4. The above graphs show the contrast in behavior between the two resins. Fitting the Ogden model to IP-PDMS curves was not possible due to the lack of yield observed. From the plot, it can be inferred that the fiber can stretch to a much greater extent, under less stress, than its IP-S counterpart.

8. Literature Review of IP-Dip

Table S1. IP-Dip Modulus Characterization from previous literature.

Citation		Test Type	Environment	Elastic Modulus (MPa)
Meza et al. (2015)	[1]	Structure Compression	SEM	2100
Oakdale et al. (2016)	[2]	Log Pile Compression	Ambient	300 – 1000 (Green)<br>400-300 (Cured)
Schroer et al. (2016)	[3]	Push-to-Pull Tensile Tester	Ambient	1500-1800 (annealed)
Bauhofer et al. (2017)	[4]	Nanoindenter	Ambient	1860 +- 150
Lemma et al. (2017)	[5]	Pillar Bending	Ambient	~750-3500
Jayne et al. (2018)	[6]	MEMs Tensile Testing	Ambient	880-960
Liu et al. (2018)	[7]	Capillary Beam Deformation	In SEM (Vacuum)	100
Bauer et al. (2019)	[8]	Tensile<br>Compressive<br>Bulk	In SEM (Vacuum)	1500-3500<br>2300+-300<br>3300
Ladner et al. (2019)	[9]	MEMs Tensile Testing	Ambient	920-3300 (Green)<br>3000-4000 (Cured)
Rohbeck et al. (2020)	[10]	Compression &<br>Tensile<br>Indention	SEM<br>SEM<br>Ambient	3200<br><br>2580+-120
Diamantopoulou et al (2021)	[11]	Compression	Ambient	1453-1741
Schweiger et al (2022)	[12]	Nano-Indentation	Ambient	1000-2100
This Study		TPP µTT	Liquid	237-591

References

[1] L. R. Meza, A. J. Zelhofer, N. Clarke, A. J. Mateos, D. M. Kochmann, J. R. Greer, *Proceedings of the National Academy of Sciences* **2015**, 112, 11502.

[2] J. S. Oakdale, J. Ye, W. L. Smith, J. Biener, *Optics express* **2016**, 24, 27077.

[3] A. Schroer, J. Bauer, R. Schwaiger, O. Kraft, *Extreme Mechanics Letters* **2016**, 8, 283.

[4] A. A. Bauhofer, S. Krödel, J. Rys, O. R. Bilal, A. Constantinescu, C. Daraio, *Advanced Materials* **2017**, 29, 1703024.

[5] E. D. Lemma, F. Rizzi, T. Dattoma, B. Spagnolo, L. Sileo, A. Qualtieri, M. De Vittorio, F. Pisanello, *IEEE transactions on nanotechnology* **2016**, 16, 23.

[6] R. K. Jayne, T. J. Stark, J. B. Reeves, D. J. Bishop, A. E. White, *Advanced Materials Technologies* **2018**, 3, 1700293.

[7] Y. Liu, J. H. Campbell, O. Stein, L. Jiang, J. Hund, Y. Lu, *Nanomaterials* **2018**, 8, 498.

[8] J. Bauer, A. Guell Izard, Y. Zhang, T. Baldacchini, L. Valdevit, *Advanced Materials Technologies* **2019**, 4, 1900146.

[9] I. S. Ladner, M. A. Cullinan, S. K. Saha, *RSC advances* **2019**, 9, 28808.

[10] N. Rohbeck, R. Ramachandramoorthy, D. Casari, P. Schürch, T. E. Edwards, L. Schilinsky, L. Philippe, J. Schwiedrzik, J. Michler, *Materials & Design* **2020**, 195, 108977.

[11] M. Diamantopoulou, N. Karathanasopoulos, D. Mohr, *Additive Manufacturing* **2021**, 47, 102266.

[12] S. Schweiger, T. Schulze, S. Schlipf, P. Reinig, H. Schenk, *Journal of Optical Microsystems* **2022**, 2, 033501.
